# Medicinal Plant Using Ground State Stabilization of Natural Antioxidant Curcumin by Keto-Enol Tautomerisation

**DOI:** 10.1007/s13659-018-0170-1

**Published:** 2018-06-22

**Authors:** S. Manimaran, K. SambathKumar, R. Gayathri, K. Raja, N. Rajkamal, M. Venkatachalapathy, G. Ravichandran, C. Lourdu EdisonRaj

**Affiliations:** 1 P.G&Research Department of Physics, Thanthai Hans Roever College (Autonomous), Perambalur, Tamil Nadu 621220 India; 2 Post Graduate and Research Department of Physics, (NANO Science Divisions), A. A. Govt. Arts College, Villupuram, Tamil Nadu 605602 India; 3 Post Graduate and Research Department of Physics, Cauvery College for Women, Tiruchirappalli, Tamil Nadu India; 4 Post Graduate and Research Department of Physics, Dr. R. K. Shanmugam College of Arts & Science, Kallakurichi, Tamil Nadu 606213 India; 5 Post Graduate and Research Department of Physics, Thiru. A. Govindasamy Govt Arts College, Tindivanam, Tamil Nadu 604002 India; 6 Post Graduate and Research Department of Chemistry, A. A. Govt. Arts College, Villupuram, Tamil Nadu 605602 India

**Keywords:** Curcumin, NMR, UV, HOMO-LUMO, MEP

## Abstract

Curcumin is a medicinal agent that exhibits anti-cancer properties and bioactive pigment in Turmeric has a huge therapeutic value. It has a keto-enol moiety that gives rise to many of its chemical properties. A recent study has shown that keto-enol tautomerisation at this moiety is implicated the effect of curcumin. The tautomerisation of curcumin in methanol, acetone and acetonitrile are used in nuclear magnetic resonance (^1^H, ^13^C) spectroscopy. It was characterized using UV, IR and Raman spectral values. The molecular electrostatic potential surface of the Curcumin has been visualized in electropositive potential in the region of the CH^3+^ group and most electronegative potential in the two oxygen atom has very strong binding group. In the following, the modality of structural and thermo dynamical parameters, electrophilicity (ω), chemical potential (μ), chemical hardness (η) and electronic charge transfer confirms the local reactivity. The rate constant of tautomerisation of curcumin shows strong temperature dependence. Molecular electrostatic potential and Temperature dependence of various thermodynamic properties like $\left( {{\hbox{C}}_{\rm{p,m}}^{0},\;{\hbox{S}}_{\rm{m}}^{0},\;{\hbox{and}}\;{\hbox{H}}_{\rm{m}}^{0} } \right)$ is increase with increase in temperature for monomer and dimer of various electrical fields.

## Introduction

Curcuma longa Linn (turmeric) is a medicinal plant botanically related to Zingaberaceae family. Turmeric powder derived from the rhizome of Curcuma longa is commonly used as a spice, food preservative and food colouring agent. It also has a long history of therapeutic uses. The compound shows yellow colour in Turmeric are three curcuminoids namely curcumin, demethoxycurcumin and bisdemethoxy curcumin. Curcumin [1,7-bis(4-hydroxy-3-methoxyphenyl)-1,6-heptadiene-3,5-dione], a yellow bioactive pigment is the major component of Turmeric. Curcumin shows some biological activities exhibiting anti-inflammatory [1–5], antifungal, anticarcinogenic, antibacterial [6], antiprotozoal wound healing, antispasmodic, anticoagulant, antitumor and hepatoprotective activities [7–10]. Antioxidant and wound-healing gives by curcumin, its anticancer and anti-viral attributes, its effect on lymphocytes, platlet aggregation, detoxification mechanism. Curcumin is a potential anticancer drug it show its effect through molecular targets and it also cure colon cancer. The water-soluble extract turmerin, inhibits HIV infected T cell proliferation. It has also been studied extensively as a chemo preventive agent in several cancer cells [11–16]. From the figure the acceptor site is the active region to cure cancer cells and also some tumors. Structurally curcuminoids are linear 1,7-diaryl-1,6-heptadiene-3,5-diones which exist in tautomeric forms as α,β unsaturated 1,3-diketo form and enol form. The proton-transfer of dissociation is associated with radical-scavenging mechanisms of curcumin. The curcumin structure containing enol form supposes to be more stable than diketo form. Curcumin has a potential singlet oxygen quencher at low conditions. The compound has an effect in protecting skin against UV light. The results indicate that unsaturation in the side chain a methoxy group on benzene ring and keto-enol in the curcumin molecule structural. The theoretical also reported wherein DFT and TD-DFT calculations help to find out keto-enol equilibrium of curcumin in solution state and low pKa value for the dissocation of enol proton. Quanta methods are useful in determining the effect of inter molecular interactions on the vibrational spectra. The pervious study explain the understanding of hydrogen bonding that conform spectroscopic approach (FT-IR, FT-Raman) with quantum chemical methods (DFT). Density Functional theory is useful in vibrational assignment, hydrogen bonding, High chemical reactivity (Homo-Lumo) and NMR. The delocalisation of π-electron is further supported by red-shifted UV–visible absorption maximum of keto-enol tautomer of curcumin.

## Analytical Instruments

The chemicals required were obtained from Sigma Aldrich chemical suppliers and are of analar grade. UV spectra were recorded on a Schimadzu UV–VIS-1601 spectrophotometer. FTIR spectra (KBr pellets) were recorded on 8101 Schimadzu FTIR spectrophotometer recorded in the region 4000–400 cm^−1^. FT-Raman spectra have been recorded in the region 3500–50 cm^−1^on a Perkin-Elmer spectrometer. The 1H NMR spectra were recorded on a Varian 300 NMR spectrophotometer. The photoluminescence spectra of curcumin deposited on SiO2/Si were recorded at room temperature on a Fluorolog-3 Model FL3-221 spectro fluorometer system (HORIBA Jobin–Yvon). The emission spectra were measured utilizing a 450 W xenon lamp as the excitation source.

## Computational Details

Advanced geometries of the title compound in the ground state were obtained by the density functional theory (DFT) [17] and B3LYP functional with 6-311++G(d,p) basis set utilizing Gaussian 09 program package. The basis set 6-311++G(d,p) fragmentation by d polarization functions on heavy atoms and p polarization functions on hydrogen atoms have been used [18–20]. Quantum theory of atoms in molecules calculations has been done to predict intermolecular interactions using this software. The conformers analysis, first hyperpolarizability, ^1^H,^13^C and ^17^O NMR, HOMO-LUMO and ESP analyses under various electric fields are carried out by B3LYP/6-311G++(d,p) method. The thermodynamic functions such as entropy, enthalpy and the heat capacity where carried out for the different temperatures. Curcumin has two hydrophobic phenyl groups. But the molecule assumes different conformations that can maximize π-π and van der Waals interactions with aromatic and some hydrophobic amino acid residues of proteins [21–25]. Phenolic hydroxyl and methoxy groups, as well as the keto and enol groups present on the ends and in the middle of the molecule has strong interaction as well as directed hydrogen-bonding.

The keto-enol tautomerism introduces additional functionality, with the possibility to locate donor and acceptor groups for hydrogen bonding in many ways. But the α,β-unsaturated keto can serve as a acceptor for nucleophilic attack. The difference between two tautomers is the hybridization of the carbon at the (α) position. While sp^2^ hybridiation is present in the keto-enol tautomer, the diketo tautomer has *sp*^3^ hybridisation. Finally, there is intramolecular hydrogen bonding in the keto-enol tautomer, which is absent in diketo tautomer of curcumin. It has been shown that the strength of the hydrogen bond at the keto-enol is strongly correlated to delocalization of π–electron in β-diketones.

## Results and Discussion

### Molecular Geometry

**Fig. 1:**
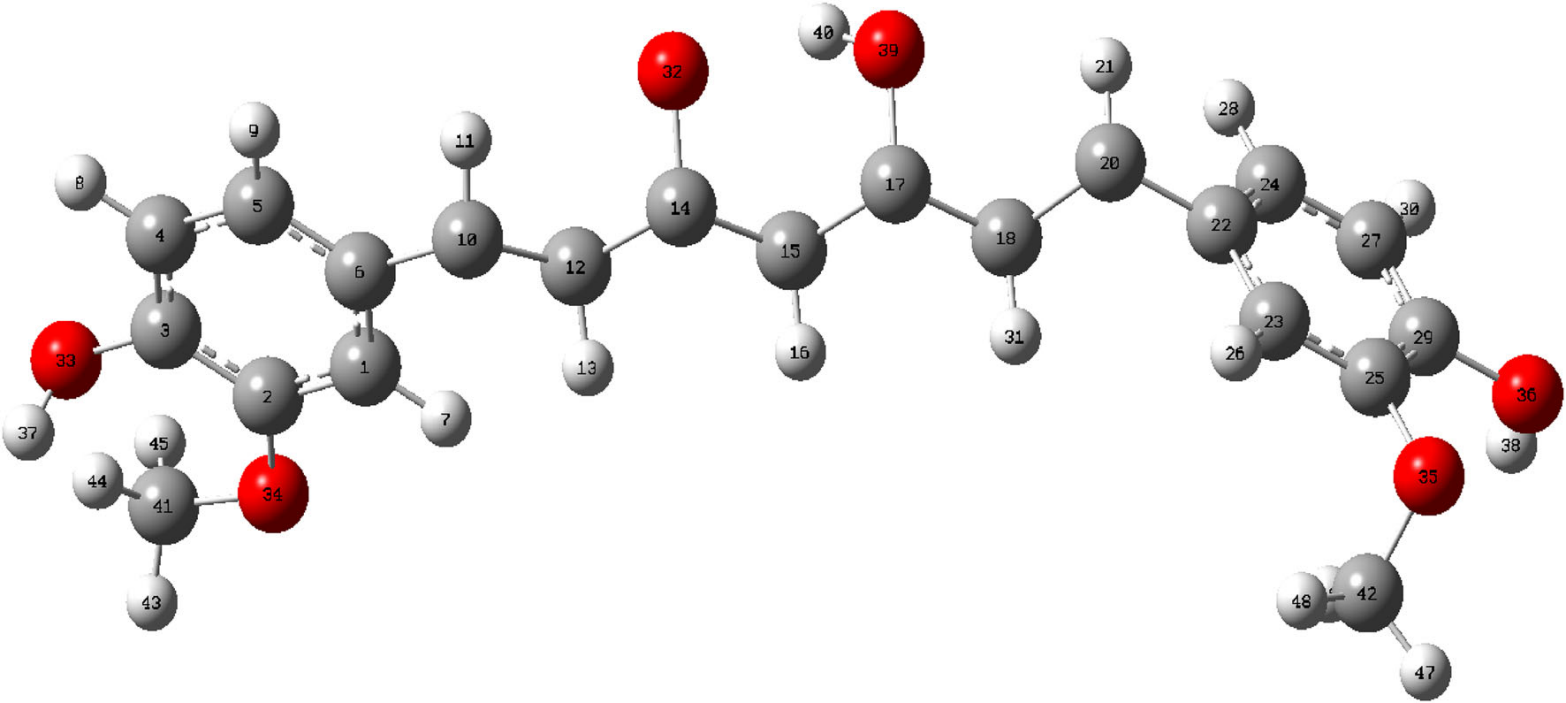
Optimized monomer molecular structure of curcumin

**Fig. 2:**
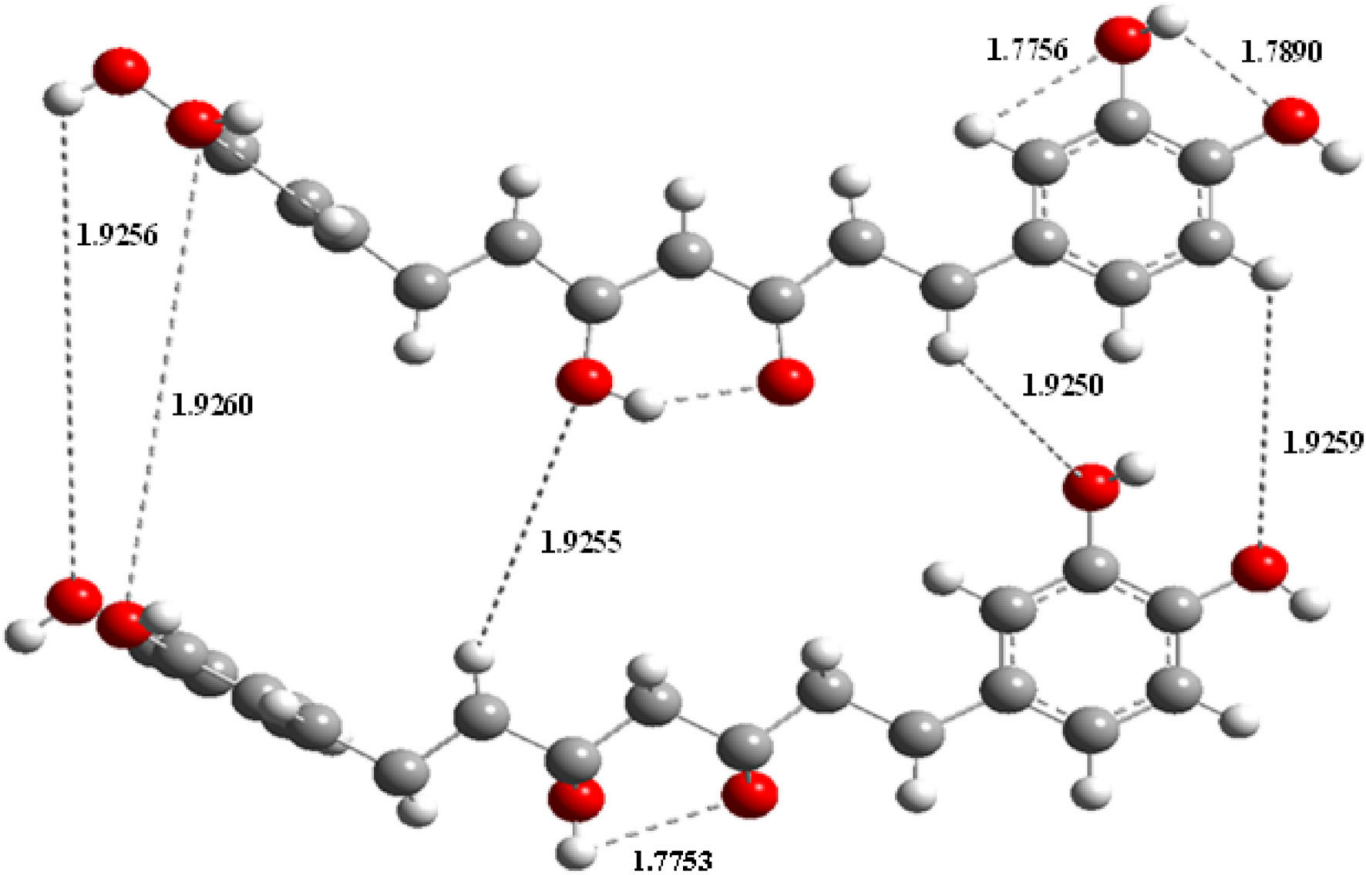
Optimized dimer molecular structure of curcumin

**Fig. 3:**
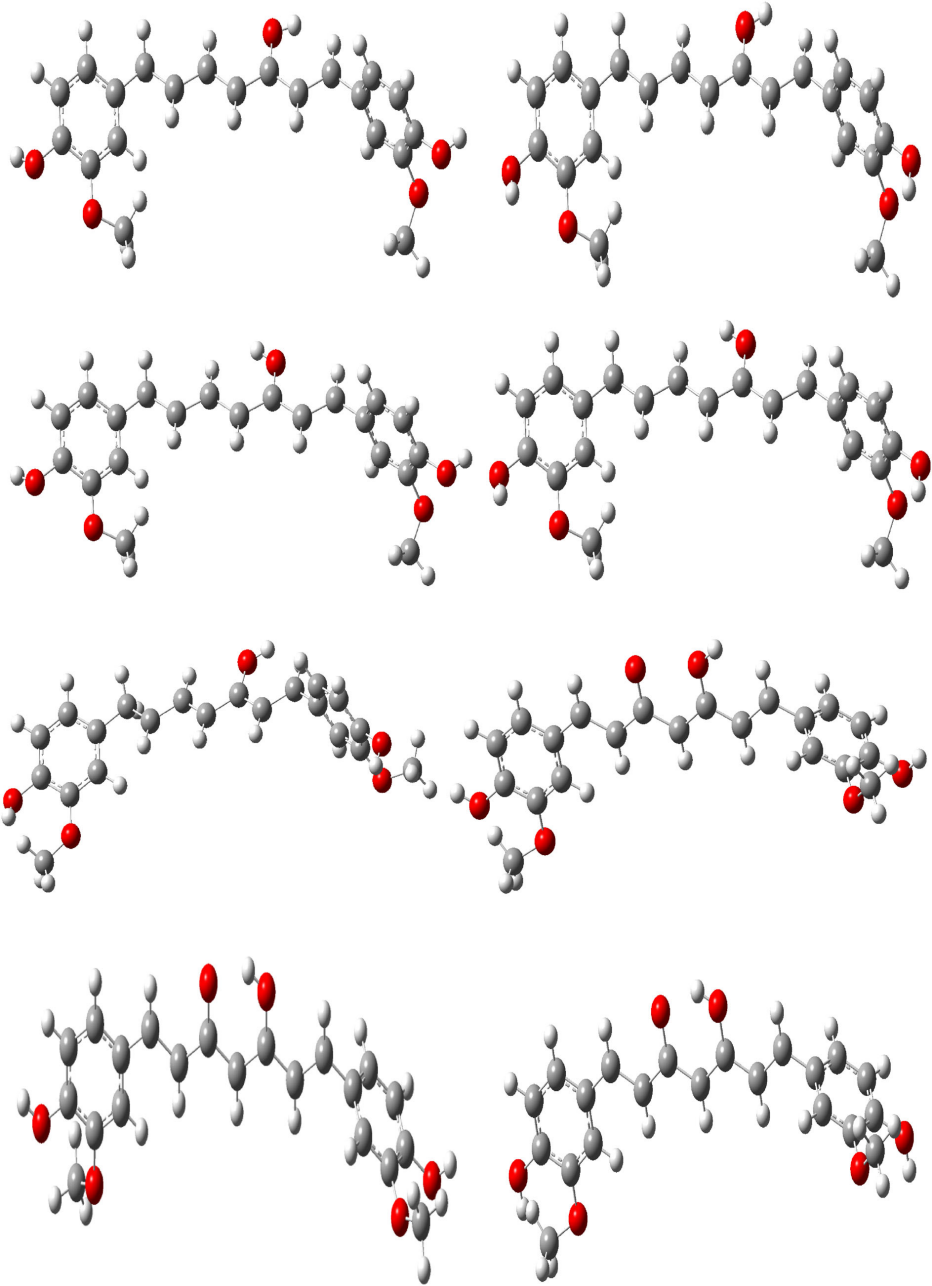

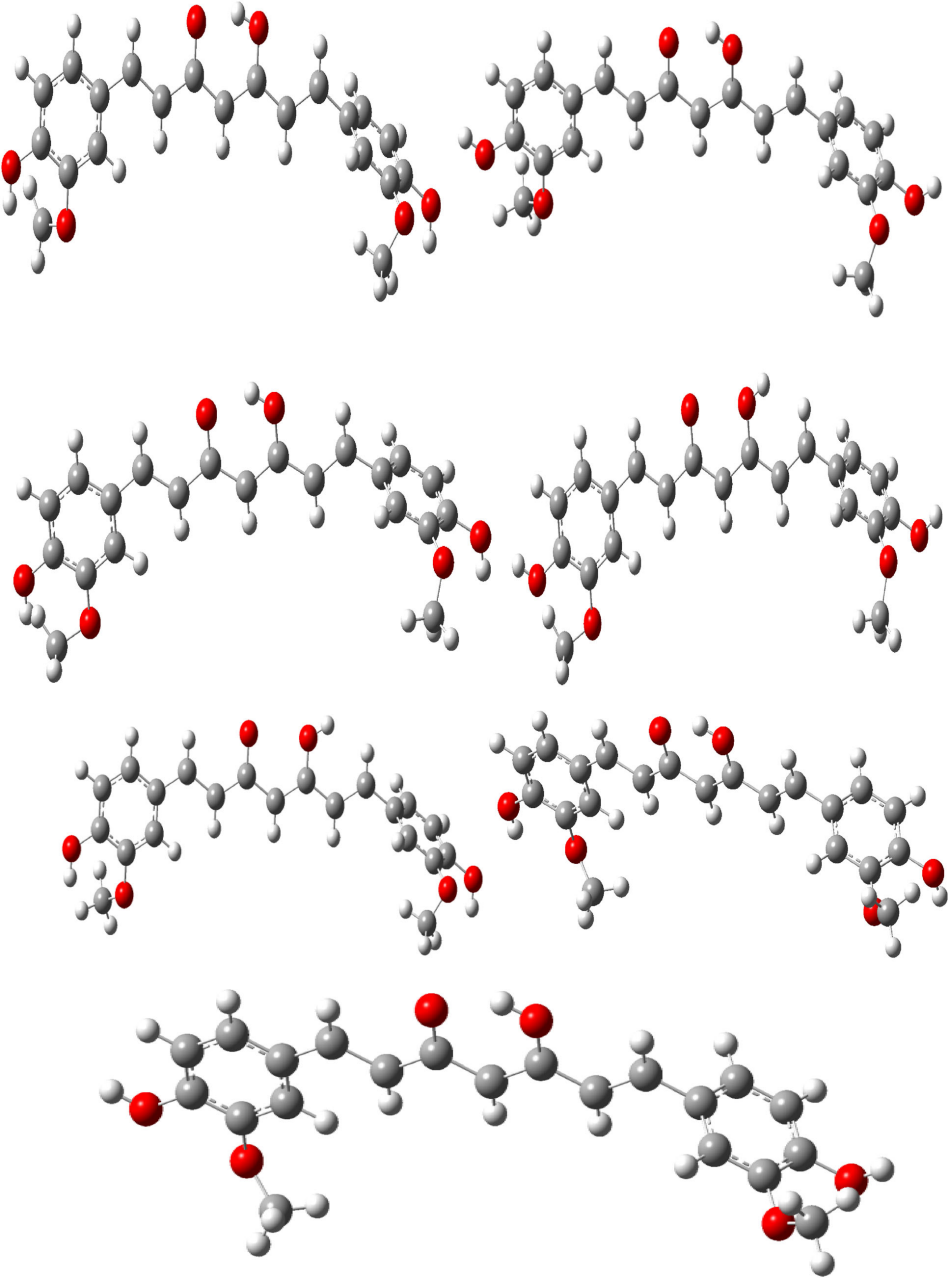
Various conformers of curcumin

Fourteen conformers are mainly expected to exist for Curcumin, respectively. The optimized parameters of monomer and dimer structures of Curcumin are represented. Generally geometrical parameters such as bond lengths, bond angles are the important factors in determining the electronic properties of the molecule that are listed in Table 1. The numbering scheme of monomer structures is denoted in Fig. 1 respectively. The optimization of dimer for the title compound is carried out in order to simulate H bonding through phenol group. The optimized structures with inter molecular hydrogen bonds description are given in Fig. 2. The intermolecular interaction is observed between C–H···O and O–H···H. This intermolecular interaction is stabilizing in Curcumin. The bond lengths of C=O are lengthened in dimer optimization [26]. To find the most optimized geometry, the energy calculations are carried out for various possible conformers are listed in Table 2. The conformers of Curcumin are shown in Fig. 3.

**Table 1:** Optimized parameters of Curcumin using B3LYP/6-311 ++G(d,p) method

Bond length	Values (Å)		Bond angle	Values (°)		Dihedral angle	Values (°)	
	Monomer	Dimer		Monomer	Dimer		Monomer	Dimer
C1–C2	1.3882	1.3920/1.3921	C2–C1–C6	132.7684	135.22/135.71	C6–C1–C2–C3	0.0651	0.0813/0.0824
C1–C6	1.411	1.4523/1.4554	C2–C1–C34	112.9745	114.21/114.22	C6–C1–C2–H7	179.9847	179.99/180.99
C1–C34	1.3865	1.3955/1.3958	C6–H1–C34	114.0209	116.13/116.14	C34–C1–C2–C3	179.7903	179.90/179.99
C2–C3	1.4157	1.4278/1.4288	C1–C2–C3	123.5507	124.86/124.89	C34–C1–C2–H7	0.0093	0.0097/0.0098
C2–H7	1.0783	1.0883/1.0893	C1–CH2–7	122.4276	123.56/123.57	C2–C1–C6–C5	− 0.02112	− 0.0253/− 0.0264
C3–C4	1.4116	1.4236/1.4246	C3–C2–H7	117.1557	119.31/119.32	C2–C1–C6–O33	179.9753	180.99/181.99
C3–C10	1.4582	1.4882/1.4892	C2–C3–C4	127.7998	128.19/128.20	C34–C1–C6–C5	178.9574	179.97/179.99
C4–C5	1.3923	1.3933/1.3933	C2–C3–C10	115.0424	117.11/117.12	C34–C1–C6–O33	− 0.0472	− 0.068/− 0.069
C4–C8	1.0822	1.0922/1.0923	C4–C3–C10	125.041	126.13/126.14	C2–C1–C34–H40	0.2032	0.5029/0.5029
C5–C6	1.3941	1.3951/1.3955	C3–C4–C5	114.4352	115.22/115.23	C6–C1–C34–H40	− 179.7731	− 179.78/− 179.79
C5–H9	1.0838	1.0839/1.0839	C3–C4–H8	120.4471	121.67/121.68	C1–C2–C3–C4	− 0.0959	− 0.099/− 0.099
C6–O33	1.3859	1.3990/1.3991	C5–C4–H8	111.9368	113.32/113.33	C1–C2–C3–C10	179.8702	179.98/179.99
C10–H11	1.088	1.098/1.0982	C4–C5–C6	130.1204	132.16/132.17	C7–C2–C3–C4	179.9817	179.99/179.99
C10–C12	1.3632	1.3720/1.3721	C4–C5–H9	117.9138	118.88/118.89	C7–C2–C3–C10	− 0.0521	− 0.524/− 0.525
C12–H13	1.0843	1.0943/1.0944	C6–C5–H9	115.1853	117.54/117.55	C2–C3–C4–C5	0.0841	0.088/0.089
C12–C14	1.4693	1.4799/1.4799	C1–C6–C5	131.1186	133.66/133.67	C2–C3–C4–O8	− 179.9804	− 179.98/− 179.99
C14–C15	1.4613	1.4931/1.4933	C1–C6–O33	113.6922	115.69/115.70	C10–C3–C4–C5	− 179.865	− 179.88/− 179.99
C14–O31	1.2728	1.2921/1.2922	C5–C6–O33	122.936	125.41/125.42	C10–C3–C4–H8	0.0405	0.042/0.045
C15–H16	1.0812	1.0922/1.0923	C3–C10–H11	118.6409	120.67/120.68	C2–C3–C10–H11	179.273	179.28/179.29
C15–C17	1.3644	1.3823/1.3824	C3–C10–C12	118.2982	119.22/119.23	C2–C3–C10–C12	177. 234	177.25/177.28
C17–C18	1.4568	1.4768/1.4769	H11–C10–C12	121.0265	122.12/122.12	C4–C3–C10–H11	− 0.7604	− 0.76/− 0.78
C17–H32	1.3902	1.3905/1.3906	C10–H12–H13	119.7609	121.23/121.23	C4–C3–C10–C12	178.9757	178.98/178.99
C18–H19	1.0807	1.0975/1.0977	C10–H12–C14	119.1506	122.50/122.51	C3–C4–C5–C6	− 0.042	− 0.045/− 0.046
C18–C20	1.3543	1.3743/1.3744	H13–H12–C14	120.3202	121.55/121.56	C3–C4–C5–C9	179.9578	179.98/179.99
C20–H21	1.0864	1.0998/1.0999	H12–C14–C15	119.0714	122.09/122.10	C8–C4–C5–C6	− 179.9677	− 179.96/− 179.99
C20–C22	1.4755	1.4957/1.4958	H12–C14–O31	120.6047	121.56/121.57	C8–C4–C5–C9	0.0321	0.0343/0.0345
C22–C23	1.4088	1.4189/1.4189	C15–C14–O31	119.9994	120.91/120.92	C4–C5–C6–C1	0.0986	0.0996/0.0998
C22–C24	1.4165	1.4177/1.4178	C14–C15–H16	121.3894	123.34/123.35	C4–C5–C6–O33	− 179.9865	− 179.98/− 179.99
C23–C25	1.3983	1.3988/1.399	C14–C15–C17	118.6033	119.60/119.61	H9–C5–C6–C1	− 179.9912	− 179.99/− 179.99
C23–C26	1.0803	1.0822/1.0823	C16–C15–C17	120.6268	121.66/121.67	H9–C5–C6–O33	0.0137	0.053/0.055
C24–C27	1.3858	1.3958/1.3959	C15–C17–C18	126.2821	128.02/128.03	C1–C6–O33–H37	179.9102	179.95/179.96
C24–H28	1.0807	1.0807/1.0809	C15–C17–H32	113.0909	115.10/115.11	C5–C6–O33–H37	− 0.0946	− 0.094/− 0.097
C25–H29	1.3877	1.3988/1.3989	C18–C17–H32	119.6991	120.22/120.21	C3–C10–C12–H13	− 179.6806	− 179.68/− 179.69
C25–H30	1.0799	1.0999/1.0999	C17–C18–H19	120.2667	121.34/121.35	C3–C10–C12–C14	− 0.1829	− 0.18/− 0.19
C27–H29	1.4081	1.4271/1.4272	H19–C18–C20	120.0295	123.12/123.13	H11–C10–C12–H13	0.0526	0.062/0.066
C27–H36	1.3982	1.3999/1.3999	C18–C20–H21	112.8729	114.56/114.57	H11–C10–C12–C14	179.5504	179.65/179.699
H29–H35	1.3816	1.3976/1.3977	C18–C20–C22	11.3221	13.56/13.57	C10–C12–C14–C15	− 178.6261	− 178.92/− 178.98
H32–O38	0.9737	0.9887/0.9899	H21–C20–C22	18.1203	19.38/19.39	C10–C12–C14–O31	1.6822	1.78/1.79
O33–H37	0.9723	0.9997/0.9998	C20–C22–C23	109.5762	119.51/119.52	H13–C12–C14–C15	0.8766	0.87/0.88
C34–H40	1.4553	1.4735/1.4736	C20–C22–C24	119.2491	119.77/119.78	H13–C12–C14–31	− 178.8151	− 178.81/− 178.89
H35–O39	0.9761	0.9961/0.9962	C23–C22–C24	11.2178	11.24/11.25	C12–C14–C15–H16	− 0.7205	− 0.75/− 0.77
H36–H41	1.4536	1.4760/1.4761	C24–C22–C25	104.7581	106.23/106.24	C12–C14–C15–C17	179.8357	179.83/179.88
H40–H42	1.0913	1.1014/1.1015	C22–C23–C25	111.1819	114.34/114.35	O31–C14–C15–H16	178.9751	178.97/178.98
H40–O43	1.0848	1.0999/1.0999	C22–C23–C26	110.1567	112.23/112.24	O31–C14–C15–C17	− 0.4687	− 0.46/− 0.49
H40–H44	1.0914	1.0966/1.0967	C25–C23–C26	109.3189	110.22/110.23	C14–C15–C17–C18	1.6541	1.65/1.67
H41–O45	1.0914	1.0978/1.0979	C22–C24–C27	110.1395	113.56/113.57	C14–C15–C17–H32	178.4673	178.46/178.55
H41–O46	1.0845	1.0950/1.0951	C22–C24–C28	110.8085	114.53/114.55	H16–C15–C17–C18	− 177.7997	− 177.79/− 177.80
H41–O47	1.0914	1.0967/1.09 68	C1–C34–H40	105.33	107.33/107.35	H16–C15–C17–H32	− 0.9865	− 0.98/− 0.99

**Table 2:** Optimized global minimum energies of different conformers of curcumin calculated at B3LYP/6− 311++G(d,p) level of theory

Conformer	B3LYP/6-311++G(d,p)
1	− 1263.12^a^
2	− 1266.90
3	− 1269.07
4	− 1264.42
5	− 1268.21
6	− 1270.88
7	− 1261.72
8	− 1260.11
9	− 1265.56
10	− 1262.22
11	− 1269.09
12	− 1267.16
13	− 1273.34
14	− 1275.06
15	− 1271.23

^a^Global minimum energy

### Two Rotor PES Scan Studies

**Fig. 4:**
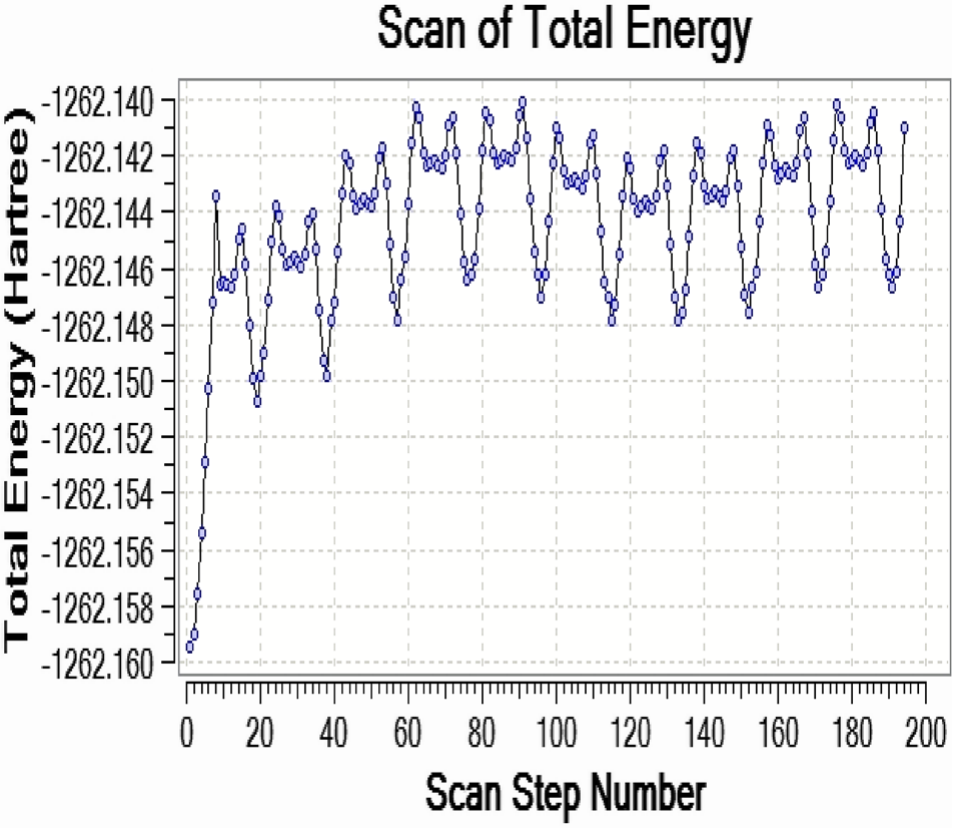
Three rotor PES Scan of curcumin

The two rotor potential energy surface (PES) scan with the B3LYP/6-311++G(d,p) level of theoretical approximations was performed for Curcumin is shown in Fig. 4, respectively. The calculated potential energy curves of the molecule in this approach a recent study were consistent with distinct minima that correspond to gauche (−)–gauche–gauche (G1gg), trans–trans–gauche (Ttg), trans–gauche–gauche (Tgg), trans–gauche–gauche (−) (Tgg1) and gauche(−)–gauche–trans (G1gt) conformers in the order of decreasing relative stability. The dihedral angle C35–C34–O6–C5 and C42–C41–O27–C24 for curcumin is also relevant coordinate for conformational flexibility within the molecule. For this rotation minimum energy curves have been obtained at 20°, 40°, 60°, 80°—as shown in Fig. 4 clearly demonstrate that flexibility inside the bonds for curcumin atoms.

## Transition State Calculations

**Fig. 5:**
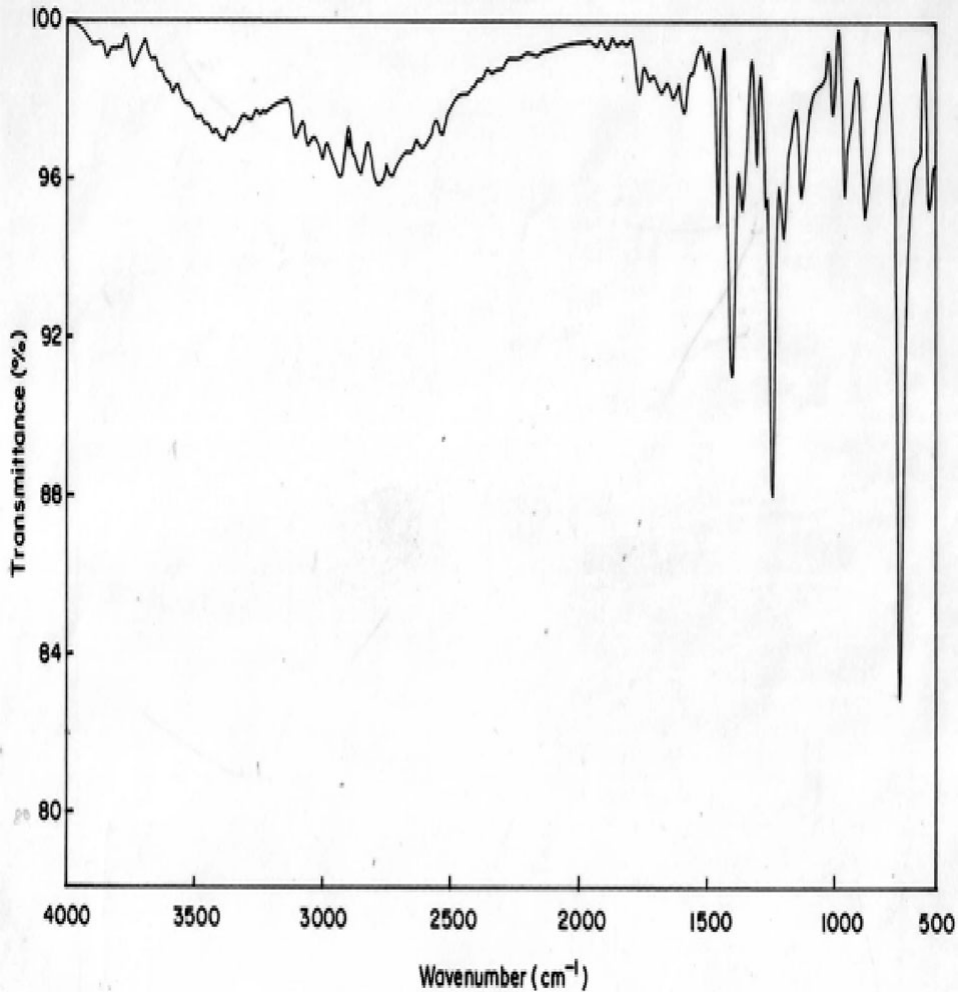
Experimental FTIR spectra of curcumin

**Fig. 6:**
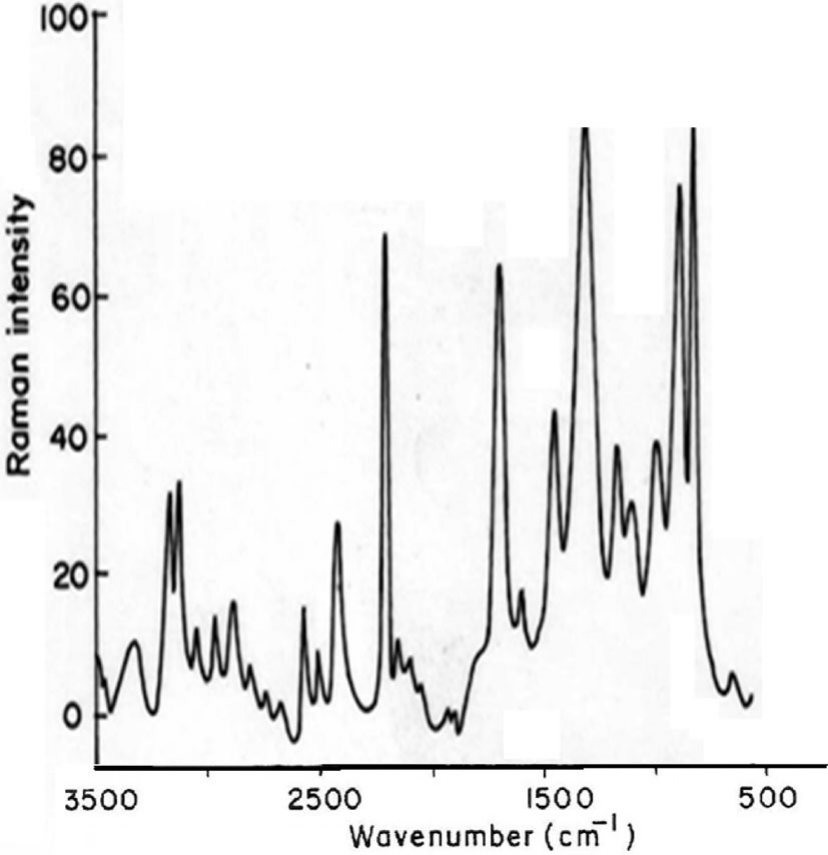
Experimental FT-Raman spectra of curcumin

The vibrational spectrum of a transition state is characterized by one imaginary frequency (implying a negative and positive field), which means that in one direction in nuclear configuration space the energy has a maximum, while in all other orthogonal directions the energy is a minimum. The fundamental modes of curcumin along with the calculated IR, Raman frequencies and normal mode descriptions (characterized by TED) are reported in Table 3. The observed and calculated FTIR and FT-Raman spectra of curcumin are performed in B3LYP levels using 6-311++G(d,p) basis set are shown in Figs. 5 and 6. Also, it should be noted that the experimental results belong to solid phase and theoretical calculations belong to gaseous phase.

**Table 3:** The observed FT-IR and FT-Raman frequencies (cm^−1^) for the Dimer, various applied electric fields (VÅ^−1^) and probable assignments (characterized by TED) of curcumin using B3LYP methods

Symmetry Species C_s_	Observed frequencies (cm^−1^)							
	FT-IR	FT-Raman	0.01 VÅ^−1^		0.02 VÅ^−1^		Dimer 0.00 VÅ^−1^	Assignments with TED (%) among types of internal co-ordinates
			Positive field	Negative field	Positive field	Negative field		
A	3308	–	3358	3196	3656	3356	4367/4113	νOH (99)
A		3289	3357	3184	3615	3351	4241/4085	νOH (98)
A	3280	3281	3346	3181	3290	3244	4222/4053	νOH (97)
A	–	3076	3342	3172	3267	3231	4217/4027	νCH (96)
A	–	3045	3338	3166	3142	3125	4188/4012	νCH (95)
A	–	3023	3331	3151	3107	3100	3654/3542	νCH (94)
A	–	2989	1632	1617	1711	1701	3007/2986	νCH (93)
A	–	2971	1591	1553	1620	1605	2840/2814	νCH (92)
A	–	2965	1553	1518	1561	1545	2734/2720	νCH (91)
A	–	2987	1527	1509	1490	1456	2717/2709	νCH (90)
A	2924	2978	1475	1461	1450	1423	2610/2605	νCH (88)
A	–	2945	1425	1415	1417	1401	2576/2554	νCH (87)
A	2900	2901	1394	1382	1329	1319	2519/2507	νCH(86)
A	–	2609	1348	1332	1279	1255	2432/2418	CH3ss (85)
A	2500	–	1340	1312	1248	1232	2409/2400	CH3ss (84)
A	–	2467	1291	1287	1205	1195	1960/1958	νCH(83)
A	–	2384	1266	1256	1170	1152	1935/1935	CH3ips (82)
A	2359	–	1206	1200	1118	1103	1925/1889	CH3ips (80)
A	2250	–	1141	1132	1075	1056	1920/1819	CH3ops(79)
A	2223	–	1123	1114	1069	1031	1895/1786	CH3ops(78)
A	2158	–	1052	1041	1020	1007	1854/1749	bOH(77)
A	–	1890	1009	1000	969	954	1780/1679	bOH(76)
A	1769	–	998	988	947	931	1631/1530	bOH(75)
A	–	1712	995	976	909	897	1617/1513	νCC (74)
A	1645	–	947	931	906	863	1402/1301	νCC(73)
A	–	1596	929	913	841	825	1203/1100	νCC (72)
A	–	1581	889	857	769	754	998/974	νCC(71)
A	1569	–	831	821	731	722	958/943	νCC (70)
A	–	1555	811	801	660	643	942/934	νCC(69)
A	1543	1544	774	753	644	622	888/853	νCC (68)
A	1522	1521	756	730	630	612	820/806	νCC(67)
A	1423	–	718	691	595	588	803/799	νC= O (66)
A	1345	–	659	635	579	563	792/743	CH_3_ ipb(64)
A	–	1289	654	616	513	500	736/721	CH_3_ ipb(63)
A	1256	–	579	534	493	475	687/604	CH_3_ sb(62)
A	–	1212	548	529	404	391	597/587	CH_3_ sb(61)
A	1178	–	499	464	378	363	534/512	CH_3_ ipr(60)
A	1156	–	438	443	353	334	487/467	CH_3_ ipr(59)
A	–	978	436	403	317	305	456/423	ωOH(58)
A	956	–	421	365	272	254	412/402	ωOH(57)
A	–	934	375	358	256	246	400/378	ωOH(56)
A	856	–	304	280	223	211	356/324	CH_3_opb(55)
A	670	–	187	156	212	207	323/312	CH_3_opb(54)
A	–	632	156	143	181	172	318/305	CH_3_opr(53)
A	614	–	122	112	153	134	300/267	CH_3_opr(52)
	–	579	109	97	137	122	281/279	CH_3_twist(51)
	540	373	87	71	110	91	236/212	CH_3_twist(50)

*ν* stretching, *ss* symmetric stretching, *ass* asymmetric stretching, *b* bending, *ω* out-of-plane bending, *R* ring, *trigd* trigonal deformation, *symd* symmetric deformation, *asymd* antisymmetric deformation, *t* torsion, *s* strong, *vs* very strong, *ms* medium strong, *w* weak, *vw* very weak

### C–H Vibrations

The aromatic organic compounds structure shows the presence of asymmetric C–H stretching vibrations in the region 3100–3000 cm^−1^ [27], which is the characteristics region for recognition on C–H stretching vibrations. In the title molecule, bands have been observed at 2924, 2900 and 3076, 3045, 3023, 2989, 2971, 2965,2987, 2978, 2945, 2901, 2467 cm^−1^ assigned to C–H stretching vibrations in FT-IR and FT-Raman spectra, respectively. The C–H in-plane and out of- plane bending vibrations normally take place as a number of intensity sharp bends in the region of 1300–1000 and 1000–750 cm^−1^, respectively. Theoretical Positive field and negative field for C–H in-plane and C–H out-of-plane bending vibrations by B3LYP/6–311++G (d, p) method showed excellent agreement with the spectrum.

### O–H Vibrations

The hydroxyl stretching vibrations are [27] observed in the region around 3500 cm^−1^. The peak is broader and its intensity is higher than that of a free O−H vibration, which indicates involvement in an inter molecular hydrogen bond. So the O−H stretching vibrations of curcumin are observed at 3308, 3280 cm^−1^ and 3289 in FT-IR and FT-Raman. The in-plane O–H deformation vibration usually appears in the region 1440–1260 cm^−1^ in the spectrum, which gets shifted to higher wave number in the presence of hydrogen bonding. The O–H out-of-plane bending vibrations give rise to broadband identified in the region 700–600 cm^−1^. Theoretical Positive field and negative field for hydroxyl vibration under goes a large shift due to hydrogen bonding.

### C–C Vibrations

The ring C–C stretching vibrations, usually occurs in the region 1400–1625 cm^−1^ [27]. In accordance with above literature data in our present study, the bands for C–C stretching vibrations are observed at 1645, 1569, 1543, 1522 cm^−1^and 1712, 1596, 1581, 1555, 1544, 1521 cm^−1^ in FT-IR and FT-Raman spectra, respectively. These observed frequencies show that, the substitutions in the ring to some extend affect the ring mode of vibrations. The comparison of the theoretically (Positive field and negative field) values are good agreement with B3LYP/6-311++G(d,p) method. The in-plane and out-of-plane bending vibrations of C–C group are also listed out in the Table 3.

### C=O Vibrations

The interaction of carbonyl group with other groups present in the system did not produce such a drastic and characteristic change in the frequency of C–O stretch as did by interaction of C–C stretch. The carbon–oxygen double bond is formed by pπ–pπ between carbon and oxygen. Because of the different electron negativities of carbon and oxygen atoms, the bonding electrons are not equally distributed between the two atoms. The lone pair of electrons on oxygen also determines the nature of the carbonyl group. The position of the C–O stretching vibration is very sensitive to various factors such as the physical state, electronic effects by substituent, ring strains. Normally carbonyl group vibrations occur in the region 1850–1600 cm^−1^ [27]. In this study, the C=O stretching vibrations of curcumin are observed at 1423 cm^−1^ in FTIR spectrum. The in-plane and out-of-plane bending vibrations of C=O group have also been identified and presented in Table 3. Positive field and negative field values are correlated with B3LYP/6-311++G(d,p) method.

### CH_3_ Group Vibrations

The curcumin molecule under consideration possesses CH_3_ groups. For the assignments of CH_3_ group frequencies one can expected that nine fundamentals can be associated to each CH_3_ group, three stretching, three bending, two rocking modes and a single torsional mode describe the methyl group. The CH_3_ symmetric stretching frequency is identified at 2500 cm^−1^ in the FTIR spectrum and 2609 cm^−1^ in the FT-Raman spectrum for curcumin. The CH_3_ in-plane stretching vibrations are identified at 2359 cm^−1^ in the FTIR spectrum and 2384 cm^−1^ in the FT-Raman spectrum. The CH_3_ symmetric bending and CH_3_ in-plane bending frequencies are attributed at 1256 and 1345 cm^−1^ in the FTIR spectrum and 1212, 1289 cm^−1^ in the FT Raman spectrum. The in-plane rocking and out-of-plane rocking modes of CH_3_ group are found at 1178, 1156 and 614 cm^−1^ in the FTIR spectrum and 632 cm^−1^ in the FT-Raman spectrum. The bands obtained at 2250, 2223 cm^−1^ and 856, 670 cm^−1^ in the FTIR spectrum for curcumin assigned to CH_3_ out-of-plane stretching and CH_3_ out-of-plane bending modes, respectively. The assignment of the bands at 540 and 579, 373 cm^−1^ in the FTIR and FT Raman spectrum for curcumin attributed to methyl twisting mode.

### NMR Data Analysis

**Fig. 7:**
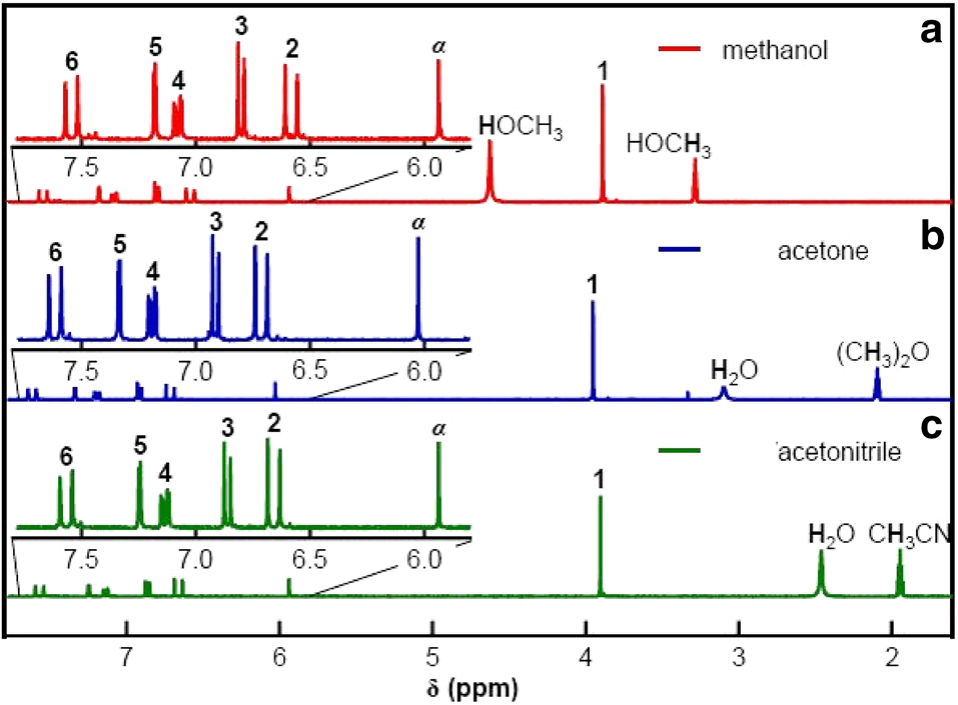
NMR spectra in **a** methanol, **b** acetone, and **c** acetonitrile

**Fig. 8:**
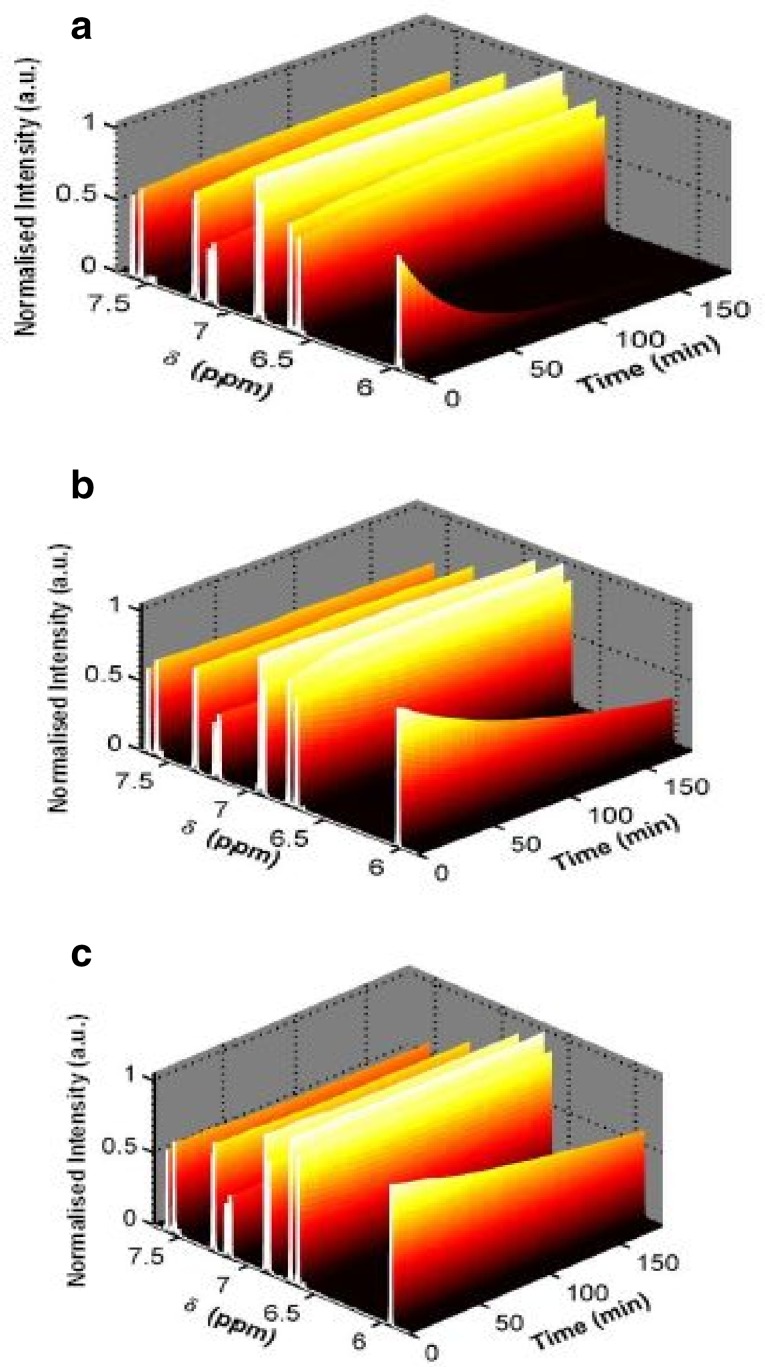
NMR spectra for curcumin at 45 °C **a** methanol, **b** acetone, and **c** acetonitrile

**Fig. 9:**
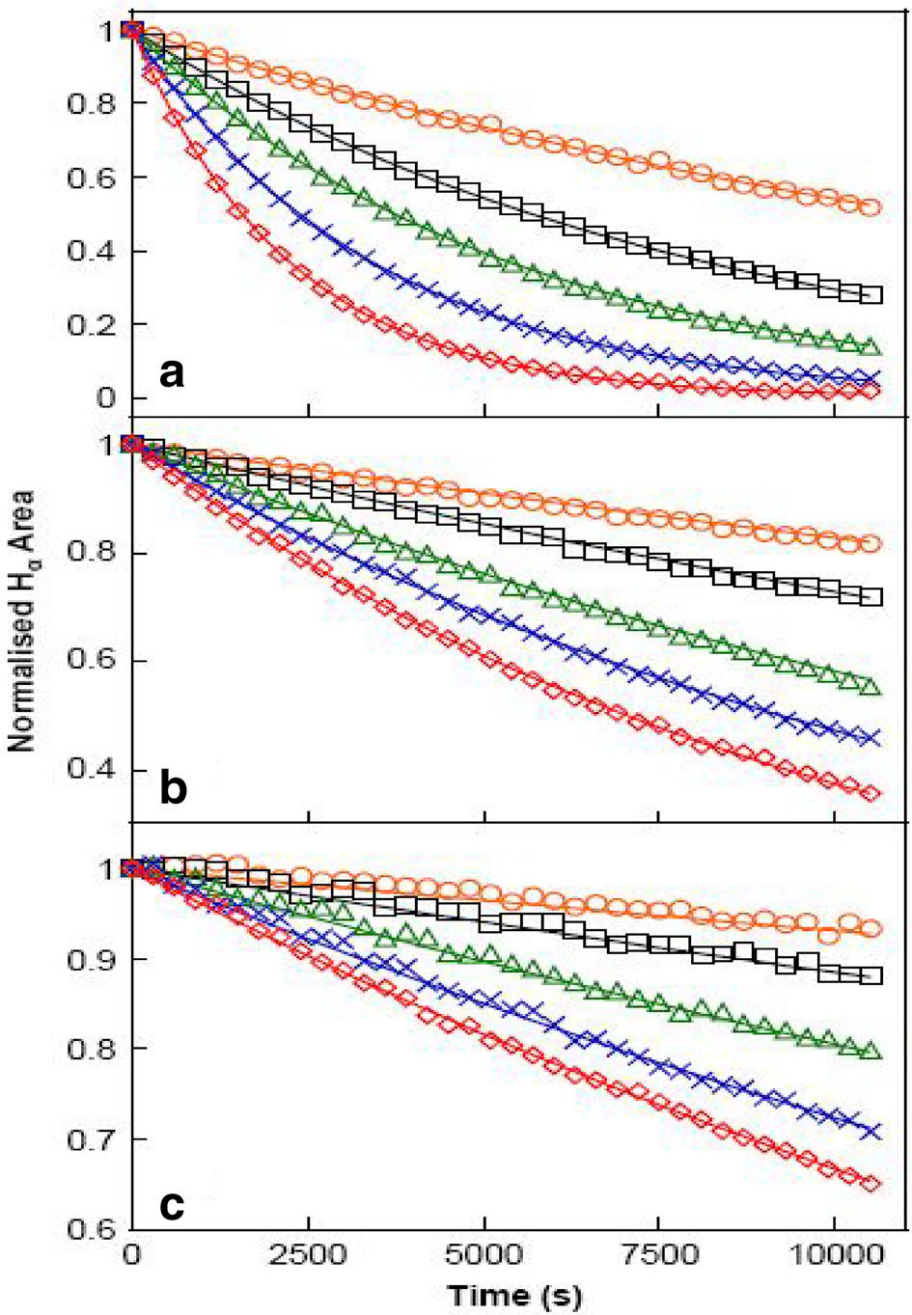
The decrease in Hα signal of curcumin in **a** methanol, **b** acetone and **c** acetonitrile as a function of time 25–45 °C. The solid curves represent the line of best-fit result of analysis with rate equations

The isotropic chemical shifts are frequently used as an aid in identification of reactive ionic species. It is acknowledged that accurate predictions of molecular geometries are essential for reliable calculations of magnetic properties. The calculated chemical shifts for ^13^C, ^1^H, and ^17^O NMR are shown in Table 4. The calculations reported here are performed in methanol solution, rather than in the gas phase, using IEF-PCM model. ^1^H atom is mostly localized on periphery of the molecules and their chemical shifts would be more susceptible to intermolecular interactions in the aqueous solutions as compared to other heavier atoms. Aromatic carbons give signals in overlapped areas of the spectrum with chemical shift values from 100 to 150 ppm [28–30]. In this study, the theoretical chemical shift values of aromatic carbons except C11 of Cur cumin are in the range of 98.3004–143.6594 ppm in the solvent. The chemical shift of C4 (143.1092 ppm in Methanol and 143.6594 ppm in Acetonitrile) is greater than the other aromatic carbons because of the substitution of OH group in Curcumin. The C25 and H32 atoms are deshielded due to the presence of electronegative oxygen in the OH group. Due to shielding effect which is the non-electronegative property of hydrogen atom, the chemical shift values of ring carbon atoms are lower than C4 and C14. The chemical shift values of aromatic protons are in the range of 6.5193–8.9553 ppm in Cur cumin for the solvents. The chemical shift value of H16 is very smaller than the aromatic protons since it is attached with electronegative atom, oxygen O31 of Cur cumin. The hydrogen atoms present in phenol group of Cur cumin experience little more shielding than other aromatic hydrogen atoms. ^17^O has a very wide chemical shift range which for small molecules partially compensates for its broad signals. The chemical shift of ^17^O is ranging from − 40 to 1120 ppm [31]. In the title compound, the peaks at 370.0681 and 367.4292 ppm are assigned to O33 which is phenol group oxygen of the methanol and acetonitrile compounds. The chemical shift magnitude of methanol and acetonitrile 209.3890 and 210.3421 ppm is indicating the presence of hydroxyl oxygen (O43) of Cur cumin. Noticeably, the oxygen chemical shift of phenol group is larger than other oxygen due to the environment. Chemical shift (δ) of the collected NMR spectra were referenced to the residue proton resonance of the corresponding solvents, which are *δ* = 3.31, 2.05, 1.94 and 3.33, 2.08, 1.99 ppm for the pentet proton signal of the two methyl group in methanol, acetone and acetonitrile, respectively. Assignments of the ^1^H NMR spectra of curcumin in methanol, acetone and acetonitrile are shown in Fig. 7. The time-dependent ^1^H NMR spectra of curcumin in methanol, acetone and acetonitrile from *δ* = 8.5 to 5.0 ppm recorded at 45 °C. It is clear that the intensity of the Hα peak, which has a *δ* of approximately 6 ppm, decrease as function of time in all three solvent. But methanol takes 3 h at 45 °C as shown in Fig. 8a and acetone, acetonitrile at 45 °C as shown in Fig. 8b, c. The Fig. 9 shows the decrease in Hα area in methanol, acetone and acetonitrile at several temperatures. The decrease in intensity of the Hα signal is proportional to the decrease of concentration of curcumin. The rate constant of tautomerisation of curcumin in methanol, acetone and acetonitrile at various temperatures are shown in Table 5. The rate constant of tautomerisation of curcumin in acetone are larger than that in acetonitrile at same temperature, indicates faster conversion of keto-enol to diketo tautomer of curcumin of acetone.

**Table 4:** The calculated ^1^H, ^11^C and ^17^O NMR isotropic chemical shifts (all values in ppm) for curcumin using GIAO method

Atoms	Theoretical method				Experimental method		
	Methanol		Acetonitrile		Methanol	Acetone	Acetonitrile
	Chemical shielding	Chemical shift	Chemical shielding	Chemical shift			
C1	29.5578	99.0123	39.5708	100.0431			
C2	33.4215	102.3436	31.1005	101.5644			
C3	38.6543	109.1278	48.5439	112.1092			
C4	40.6123	143.1092	45.2344	140.6594			
C5	66.2360	123.3412	66.2360	125.3789			
C6	76.1278	130.5678	79.2070	133.7841			
H7	23.0591	6.5193	25.9001	6.9900	5.06	6.36	8.08
H8	79.3412	6.9356	88.4629	7.1991	5.70	6.95	7.92
H9	80.4576	7.5067	90.7456	7.8903	6.26	7.12	7.16
C10	123.4390	133.2378	135.9340	121.8759			
H11	55.7123	6.7953	59.1239	6.9137	5.59	7.04	7.77
C12	122.5634	121.2376	120.3454	120.346			
H13	61.7823	8.0551	60.8203	8.9553	5.92	6.61	7.91
C14	134.7654	119.4367	139.6050	121.2310			
C15	120.7720	110.5476	121.2770	112.6754			
H16	78.3120	7.9345	78.3120	8.0098	6.85	6.50	8.12
C17	136.8976	99.2310	126.8116	110.4328			
C18	129.3245	78.3451	120.3512	99.4512			
H19	88.2786	8.6130	80.7806	8.9973	6.19	7.34	8.34
C20	132.5431	77.8907	130.5301	87.9832			
H21	87.9234	127.8923	90.2304	119.8235	5.88	7.77	8.59
C22	128.9812	122.3056	118.8129	116.2389			
C23	125.7235	115.4523	122.3545	120.4521			
C24	126.6709	111.2326	125.7091	115.2315			
C25	132.2310	109.4376	130.3106	110.3678			
C26	129.9076	103.3754	130.9006	106.2312			
C27	122.6534	110.2783	125.3423	115.6754			
H28	88.23678	7.9913	90.2780	8.6593	6.74	7.09	8.10
H29	67.9864	6.8023	65.6488	6.9993	6.47	6.23	8.25
H30	60.5431	7.1123	69.4112	7.5103	6.90	6.65	8.67
O31	214.9271	102.5643	215.910	111.2312			
H32	88.2390	6.9930	80.3910	7.2345			
O33	218.9661	109.2165	210.9610	10.3267			
C34	136.8126	127.7612	130.8113	120.4321	3.31	2.05	1.94
H35	80.8257	8.0987	88.8723	8.5930			
H36	44.6512	8.4436	48.5192	8.5193			
H37	49.0378	8.0987	55.0845	8.2305			
O38	219.1004	222.4512	224.0456	220.2312			
C39	121.2786	103.5643	125.6234	115.5409	3.33	2.08	1.99
H40	55.8762	8.1913	58.7961	8.5123			
H41	58.9064	7.4536	54.6674	7.536			
H42	65.8924	7.7776	75.8490	7.9983			
O43	221.1765	209.3890	219.1534	210.3421			
H44	69.7865	8.0674	61.8605	8.5110			
O45	225.2564	201.3267	205.6004	222.3267			
O46	229.6054	221.4512	219.0540	220.7634			
H47	72.3490	7.9876	75.9090	8.4593

**Table 5:** Rate constant of tautomerisation of curcumin in methanol, acetone and acetonitrile several temperatures

Temperature	K	Methanol	Acetone	Acetonitrile
298	1000	12.0 ± 1.9	4.0 ± 0.3	1.7 ± 0.3
303	890	20.9 ± 6.9	6.2 ± 0.9	2.6 ± 1.5
308	800	36.8 ± 2.5	11.10 ± 0.8	4.2 ± 0.9
313	720	55.2 ± 3.1	12.9 ± 1.6	8.1 ± 2.2
318	650	88.1 ± 10.1	18.9 ± 1.7	8.9 ± 3.1

### UV–Vis Spectral Analysis

**Fig. 10:**
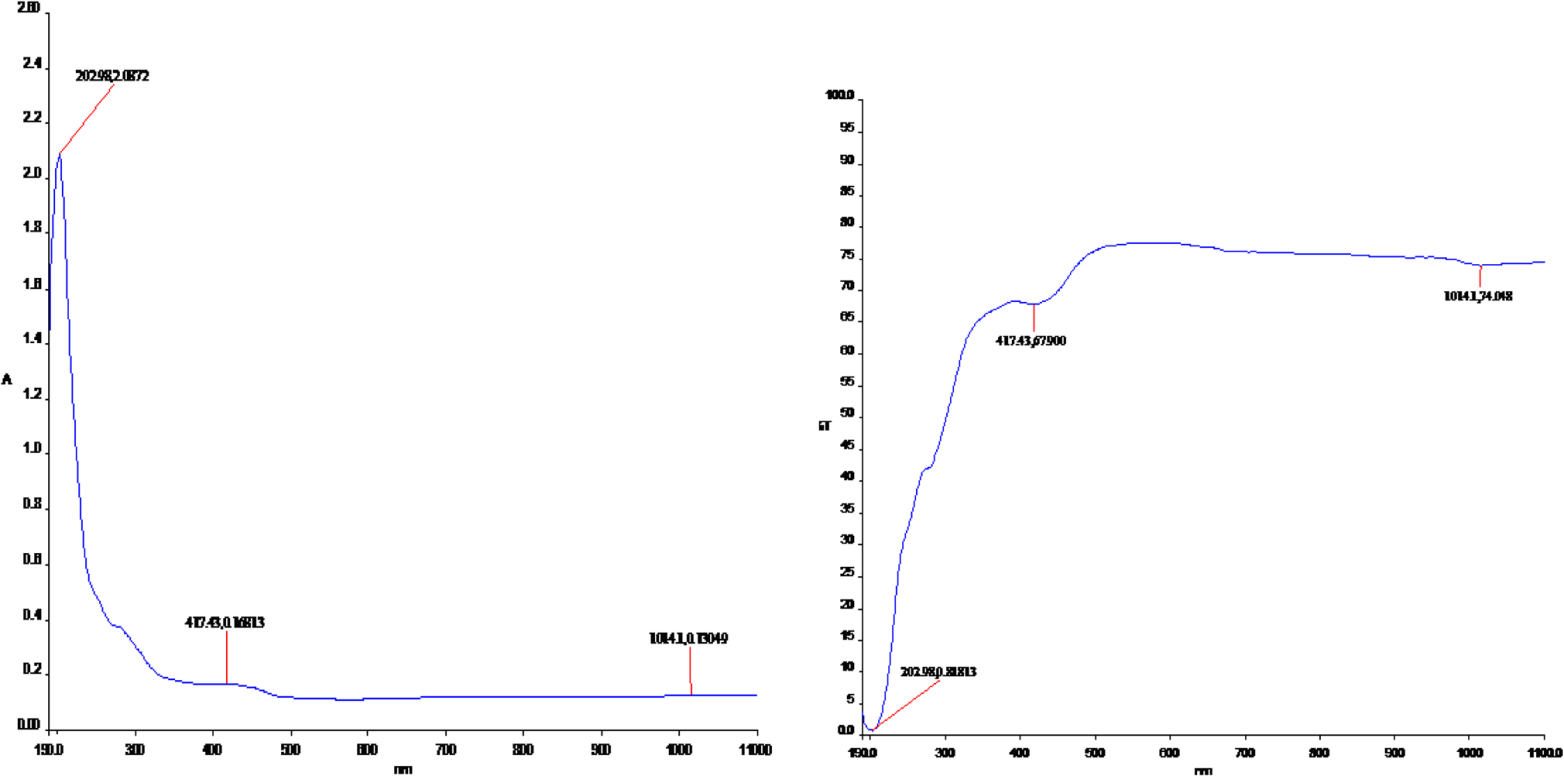
Experimental absorbance and transmittance UV–Vis spectra of curcumin

The visible and UV spectra of curcumin represent transitions between electronic energy levels. The transitions between a bonding or lone-pair orbital are non-bonding or anti-bonding orbital. But the absorption bandwidth could indicate the degree of agglomeration [32]. So the electronic absorption corresponds to the transition from the ground to the first excited state and is mainly described by one electron excitation from the highest occupied molecular orbital (HOMO) to the lowest unoccupied molecular orbital (LUMO).Electronic transitions are usually classified according to the orbitals engaged or to specific parts of the molecule involved. Common types of electronic transitions in organic molecules are π-π*, n-π* and π*(acceptor)-π (donor). For Curcumin the UV–Vis absorption and transmisson spectra have been studied and show three intense peaks of 202, 417, 1014 nm for Ethanol and THF in Table 6. Observed band at 417 nm is due to the π-π* transition. The less intense band centered at 202 nm is due to the partly forbidden n-π* transition. The more intense band observed at 1014 nm is described to an allowed π*- π transition. Based on the above photo absorption theory, the frontier molecular orbitals (FMO_S_) of the molecule have been investigated according to the results obtained from the B3LYP/6-311++G(d,p) calculations. The experimental absorbance and transmittance UV–Vis spectra are shown in Fig. 10. It also gives us the hint that the charge recombination should also be taken into account besides the widely considered criteria to design and screen of new efficient molecule for experimentalist.

**Table 6:** The computed excitation energies, oscillator strength, electronic transition configuration wavelength of Curcumin using TD-DFT/B3LYP/6-311++G(d,p)

Theoretical method			Transition with contribution	Theoretical method			Transition with contribution	Experimental method	
Ethanol				THF					
EE (ev)	Oscillator strength *f*	Wavelength (nm)		EE (ev)	Oscillator strength *f*	Wavelength (nm)		Ethanol (nm)	THF (nm)
0.0972	0.0076	1279.35	H-1 → L (59.44%)	0.0972	0.0052	1398. 53	H-1 → L (69.24%)	202	202
1.3777	0.0102	899.94	H → L+1 (50.84%)	1.3777	0.0309	1009. 32	H → L+1 (49.76%)	417	417
1.4709	0.0788	842.93	H → L (64.52%)	1.4709	0.0523	947. 30	H → L (56.12%)	1014	1014
1.9432	0.0892	957.56	H-1 → L (44.52%)	1.9432	0.0690	910.53	H → L+1 (70.28%)		
2.7658	0.0643	912.70	H → L+1 (75.14%)	2.7658	0.0765	999.22	H-1 → L (42.18%)		
2.0971	0.5625	785.88	H-2 → L (61.72%)	2.0971	0.896	845.36	H → L (77.57%)

### Transport Properties

**Fig. 11:**
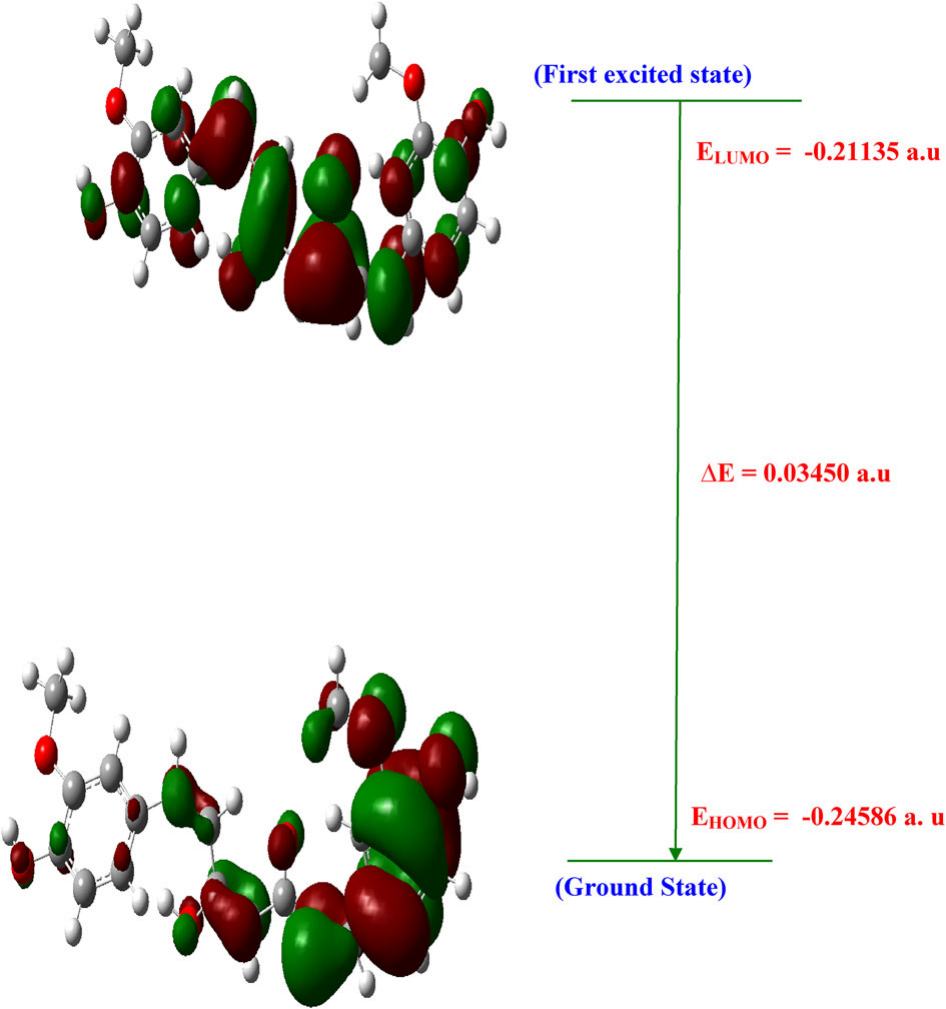
HOMO-LUMO plot of curcumin

The spatial distribution of the frontier orbital is a three dimensional representation of local electron density of the molecule. The highest occupied molecular orbital (HOMO), the lowest unoccupied molecular orbital (LUMO) are the frontier orbital’s, the difference between them are known as HOMO–LUMO gap (HLG). HLG determines the transport properties of the molecule. Large decrease in the HLG predicts the possibility of having reasonable conduction through the molecule, since, the conductivity increases with decreases in HLG. So the field increases (0.00–0.15 VÅ ^−1^) the HLG extensively decreases by using Gaussian 09 programme package. Figure 11 illustrates the spatial distribution of the molecular orbital of the molecule. Table 7, shows the small HLG exists in this molecular system, the possibility of conduction through the molecules is found to be very less, therefore, it almost act as an insulator.

**Table 7:** Electronic properties with various electric field’s of curcumin

Parameters	0.00 VÅ^−1^	0.05 VÅ^−1^	0.1 VÅ^−1^	0.15 VÅ^−1^
HOMO (a.u)	− 0.256717	− 0.234112	− 0.232379	− 0.245860
LUMO (a.u)	− 0.189864	− 0.170982	− 0.206169	− 0.211352
Energy gap (Eg) (a.u)	0.066853	0.06313	0.02621	0.034508
Chemical hardness(η) (a.u)	0.066853	0.06313	0.02621	0.034508
Chemical potential (µ) (a.u)	− 0.223291	− 0.202547	− 0.219274	− 0.228606
Electronegativity (χ) (a.u)	+ 0.223291	+ 0.202547	+ 0.219274	+ 0.228606
Softness(S) (a.u)	14.958192	15.84033	38.15338	28.97879
Electrophilicity index (ω) (a.u)	0.372898	0.32493	0.91723	0.75723

#### Electric Fields on HOMO–LUMO Analysis

**Fig. 12:**
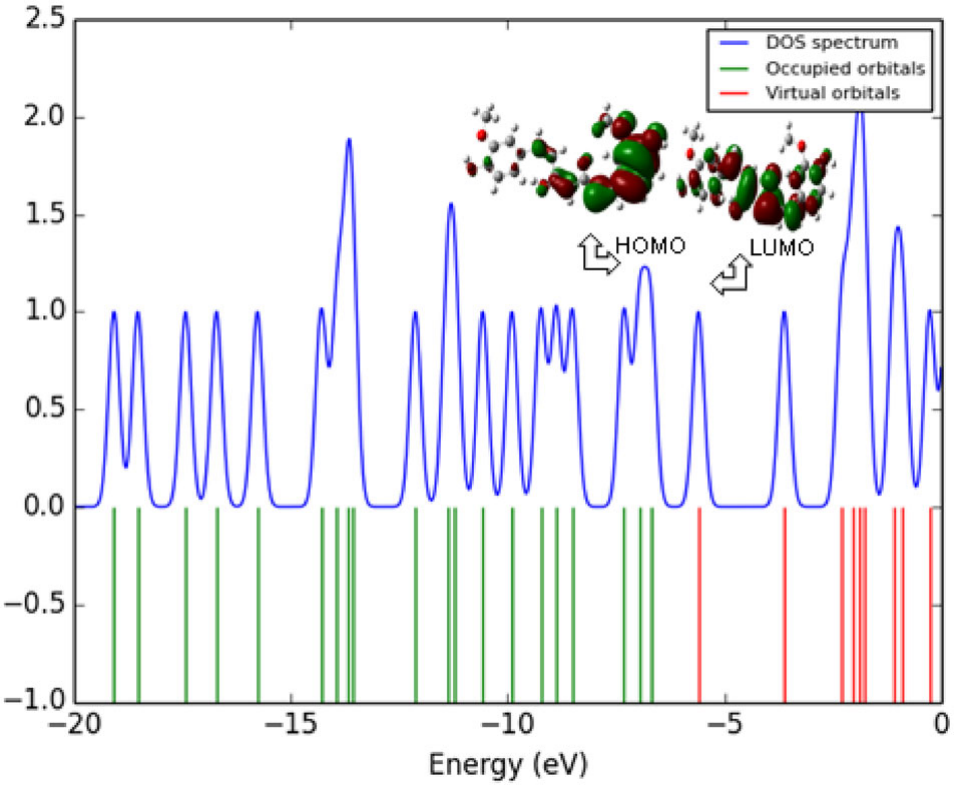
DOS spectrum of curcumin

The analysis of the wave function is mainly described by one-electron excitation from the highest occupied molecular orbital (HOMO) to the lowest unoccupied molecular orbital. HOMO–LUMO analysis of these compound is done at B3LYP/6- 311++G(d,p) level of theory for the zero field and fields of 0.05.01 and 0.15 VA^−1^. Figure 11 illustrates the orbital distributions of HOMO and LUMO levels of the title compound for the zero field and biasing steps of 0.05.01 and 0.15 VA^−1^. HOMO is delocalized mainly on ring carbons; the oxygen of hydroxy also takes part in the formation of HOMO of curcumin for zero fields. The strong electron withdrawing group O of polyphenol group attracts the charge density while on other hand electron donating group hydroxy is attached on left hand of curcumin which would strengthen the donor ability. Hence LUMOs are distributed on the polyphenol group. In Curcumin, HOMO is delocalized mainly on ring carbons and there is no distribution of HOMO on polyphenol group. Figure 11 shows that there is no electronic projection in HOMO and LUMO over the ring hydrogen atoms of the compound in zero fields. The values of HOMO energy, LUMO energy and HLG are used as an indicator of kinetic stability of the molecule. Which shows that substituted hydroxyl have no effect on the title compound. When the field increases (0.0–0.15 VA^−1^), the HOMO–LUMO gap (HLG) extensively decreases from 0.06853 to 0.0345 eV for curcumin, respectively (Table 7). This large decrease in the HLG implies that the possibility of having reasonable conduction through the molecule, hence the conductivity increases with decreases in HLG. The variation of HLG with applied the electric field is also verified with density of states (DOS) spectrum. Figure 12 shows the density of states (DOS) for zero field, 0.05, 0.1 and 0.15 VA^−1^.The green and red lines in the DOS spectrum are indicating that the HOMO and LUMO levels. The value of HLG measured in DOS spectrum is almost the same with the calculated value by Gaussian 09 W program package [33–35]. The DOS spectrum also shows that HOMO–LUMO gap decreases as increase in electric field. Further insight into the chemical reactivity analysis in relation to their electronegativity, chemical hardness and their aromaticity difference would assess the suitability of application process in curcumin.

### Mullliken Charges

**Fig. 13:**
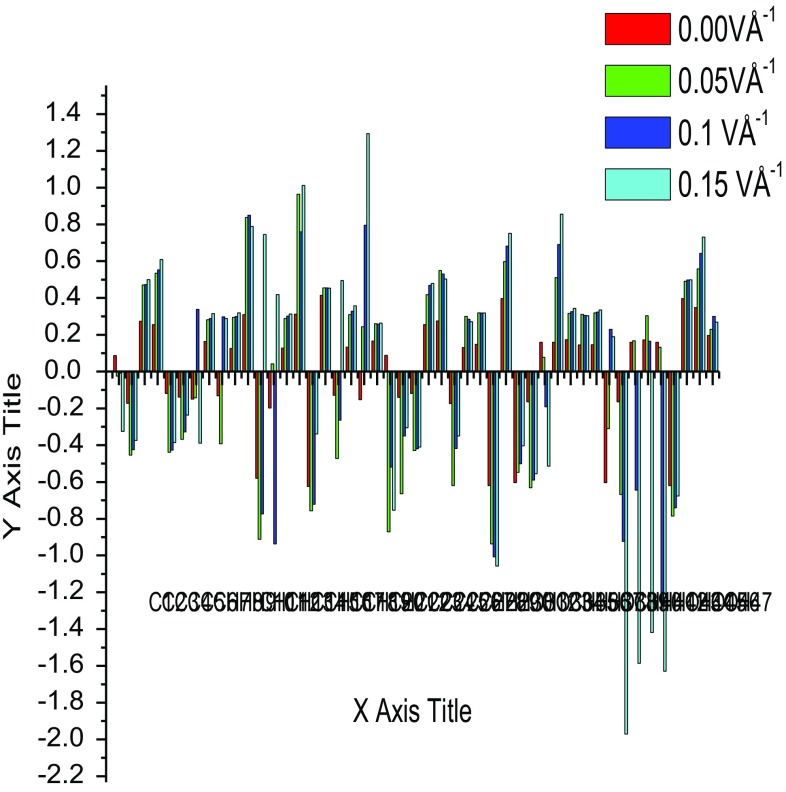
Mulliken plot of curcumin

The Mulliken psychoanalysis is the finest population analysis method. The electron population of each atom of the molecules is identifying, because of calculating the mulliken charges are explained by the density functional methods. The charge distribution of the Curcumin gives the Carbon and Hydrogen charges had both signs. Oxygen atoms have negative charges in all basic sets, which are donor atoms. Mulliken atomic charge computation play a vital role in the relevance of quantum chemical calculation to molecular system because of atomic charge effect, dipole moment, molecular polarizability, electronic structure of the molecular systems. By using the Gaussian output shown in the Table 8. The molecule has different charge distribution with respect to the dynamic basic sets for quantum calculations. The predicted atomic charges are in graphical representation (Fig. 13). To change the basic set, the charge distribution should be changed. The charge change with origin set due to polarization For illustration, the charge of H(16) and H(19) atoms are -0.3390e, 0.3577e at B3LYP/6-311+G(d,p) in the title compound curcumin. Thus the charges of the atoms are not varied according to electric field. Charge migration to heavy atoms can be related to molecular interactions in curcumin molecules.

**Table 8:** Mulliken charge of curcumin using B3LYP/6-311 ++G(d,p)

Atom No.	0.00 VÅ^−1^	0.05 VÅ^−1^	0.1 VÅ^−1^	0.15 VÅ^−1^
C1	0.086847	− 0.024182	− 0.008302	− 0.325170
C2	− 0.172636	− 0.454099	− 0.424706	− 0.375011
C3	0.274180	0.470479	0.472686	0.499956
C4	0.254393	0.533688	0.551875	0.609097
C5	− 0.118631	− 0.438842	− 0.426789	− 0.386371
C6	− 0.138775	− 0.368391	− 0.328251	− 0.236610
H7	− 0.149836	− 0.142547	0.337729	− 0.390048
H8	0.163524	0.280981	0.287917	0.314909
H9	− 0.131957	− 0.392618	0.297198	0.288442
C10	0.124668	0.294558	0.298511	0.318498
H11	0.308963	0.836655	0.849524	0.788269
C12	− 0.580530	− 0.912246	− 0.774070	0.745018
H13	− 0.198208	0.041912	− 0.936011	0.418385
C14	0.127441	0.288061	0.300280	0.312129
C15	0.311387	0.964421	0.759970	1.011231
H16	− 0.625503	− 0.756989	− 0.721159	− 0.339039
C17	0.414234	0.454330	0.454291	0.453960
C18	− 0.128214	− 0.472383	− 0.264393	0.494700
H19	0.132174	0.309119	0.327345	0.357721
C20	− 0.153115	0.242813	0.795146	1.293723
H21	0.165946	0.260296	0.256487	0.263050
C22	0.087479	− 0.871133	− 0.519403	− 0.753220
C23	− 0.140098	− 0.663842	− 0.349952	− 0.304823
C24	− 0.118493	− 0.429575	− 0.419701	− 0.409819
C25	0.254199	0.417244	0.469102	0.478777
C26	0.274591	0.548879	0.531076	0.503005
C27	− 0.173794	− 0.620065	− 0.418024	− 0.349877
H28	0.131018	0.299944	0.284802	0.270245
H29	0.147718	0.318470	0.317818	0.318071
H30	− 0.619578	− 0.936813	− 1.006706	− 1.057375
O31	0.397470	0.597232	0.681999	0.750933
H32	− 0.603827	− 0.547544	− 0.500546	− 0.403394
O33	− 0.164927	− 0.631759	− 0.589280	− 0.554964
C34	0.159547	0.076608	− 0.191618	− 0.513757
H35	0.159548	0.510890	0.690412	0.854806
H36	0.172965	0.315799	0.325133	0.343978
H37	0.144435	0.310030	0.304759	0.302369
O38	0.145587	0.318952	0.323437	0.335420
C39	− 0.604032	− 0.309873	0.230250	0.189667
H40	− 0.164949	− 0.667438	− 0.922367	− 1.971154
H41	0.159706	0.166929	− 0.644723	− 1.586309
H42	0.172648	0.302583	0.164796	− 1.418660
O43	0.159708	0.131597	− 1.275581	− 1.628964
H44	− 0.619699	− 0.786000	− 0.740768	− 0.675913
O45	0.397439	0.490274	0.497844	0.498682
O46	0.347474	0.557234	0.641990	0.730936
H47	0.195957	0.230299	0.299480	0.268850

### Nonlinear Optical Properties

The hyperpolarizability (β), dipole moment (µ) and polarizability (α) were calculated using B3LYPmethod with 6-311++G(d,p) basis set on the basis of the finite-field approach. The complete equations for calculating the magnitude of total static dipole moment (µ), Vector-first hyperpolarizability (β_vec_), the anisotropy of the polarizability (Δα) and the First hyperpolarizability (β), using the x, y, z components from Gaussian 09 W output is as follows:

$$ {\text{E}} = {\text{ E}}_{0} {-}\upmu_{\alpha } {\text{F}}_{\alpha } {-} 1/ 2\alpha_{\alpha \upbeta } {\text{F}}_{\alpha } {\text{F}}_{\upbeta } {-} 1/ 6\upbeta_{\alpha } \upbeta_{\gamma } {\text{F}}_{\alpha } {\text{F}}_{\upbeta } {\text{F}}_{\gamma } + \ldots \ldots $$

where E_0_ is the energy of the unperturbed molecule, F_α_ is the field at the origin, and μ_α_, ααβ and βαβγ are the components of dipole moment, polarizability and the first order hyperpolarizabilities, respαectively [36]. The total static dipole moment μ, and the mean first hyperpolarizability (β) using the x, y, z components, they are defined as:

$$ \upmu = \, \left( {\upmu_{\text{x}}^{2} + \upmu_{\text{y}}^{2} + \upmu_{\text{z}}^{2} } \right)^{1/2} $$

$$ \upbeta = \, \left( {\upbeta_{\text{x}}^{2} + \upbeta_{\text{y}}^{2}+ \upbeta_{\text{z}}^{2} } \right)^{1/2} $$

where

$$ \upbeta_{\text{x}} = \upbeta_{\text{xxx}} + \upbeta_{\text{xyy}} + \upbeta_{\text{xzz}} $$

$$ \upbeta_{\text{y}} = \upbeta_{\text{yyy}} + \upbeta_{\text{xxy}} + \upbeta_{\text{yzz}} $$

$$ \upbeta_{\text{z}} = \upbeta_{\text{zzz}} + \upbeta_{\text{xxz}} + \upbeta_{\text{yyz}} $$

The first hyperpolarizability is calculated using B3LYP/6-311G++(d,p) method. Since the value of hyperpolarizability (β) of the Gaussian 09 W output is reported in atomic units (a.u.), the calculated values have been converted into electrostatic units (esu) (β: 1 a.u. = 8.639 × 10^−33^ esu). The first hyperpolarizabilities of curcumin are 3.0450 × 10^−30^ esu, respectively and are presented in Table 9. The first hyperpolarizability values of title compound are greater than that of urea (μ and β of urea are 1.3732 Debye and 0.3728 × 10^−30^ esu obtained by B3LYP/6-311++G(d,p) method). Comparatively, first hyperpolarizability shows that curcumin is suitable for nonlinear optical studies.

**Table 9:** Nonlinear optical properties of curcumin at B3LYP/6-311++G(d,p) methods and basis set calculations

NLO behaviour	B3LYP/6-311++G(d,p)
Dipole moment (μ)	3.1727 Debye
Mean polarizability (α)	1.3793 × 10^−30^ esu
Anisotropy of the polarizabilty (Δ_α_)	3.2723 × 10^−30^ esu
First hyperpolarizability (β)	3.0450 × 10^−30^ esu
Vector-first hyperpolarizability (β_vec_)	1.8270 × 10^−30^ esu

### Molecular Electrostatic Potential (MEP)

**Fig. 14:**
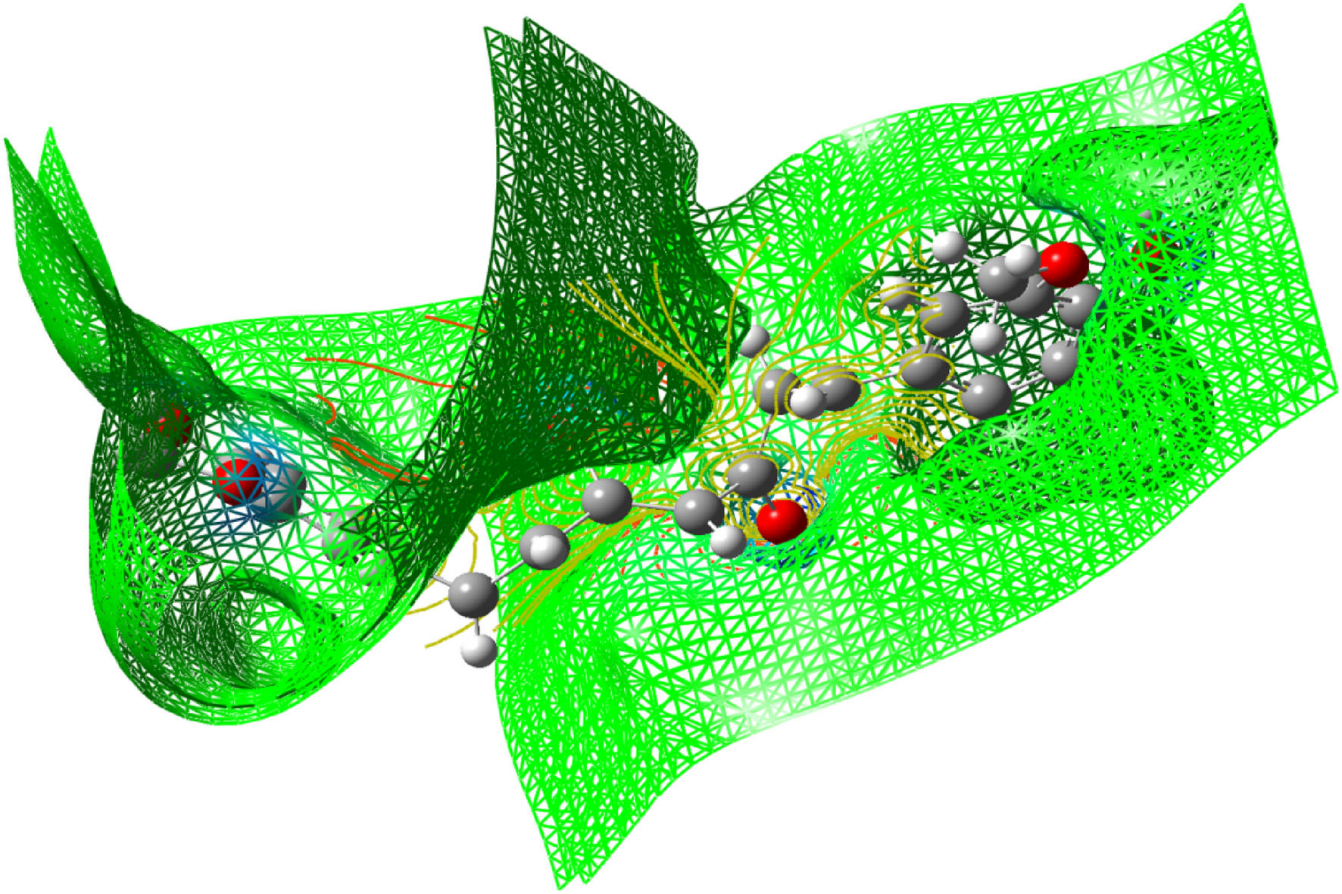
The total electron density surface of curcumin

**Fig. 15:**
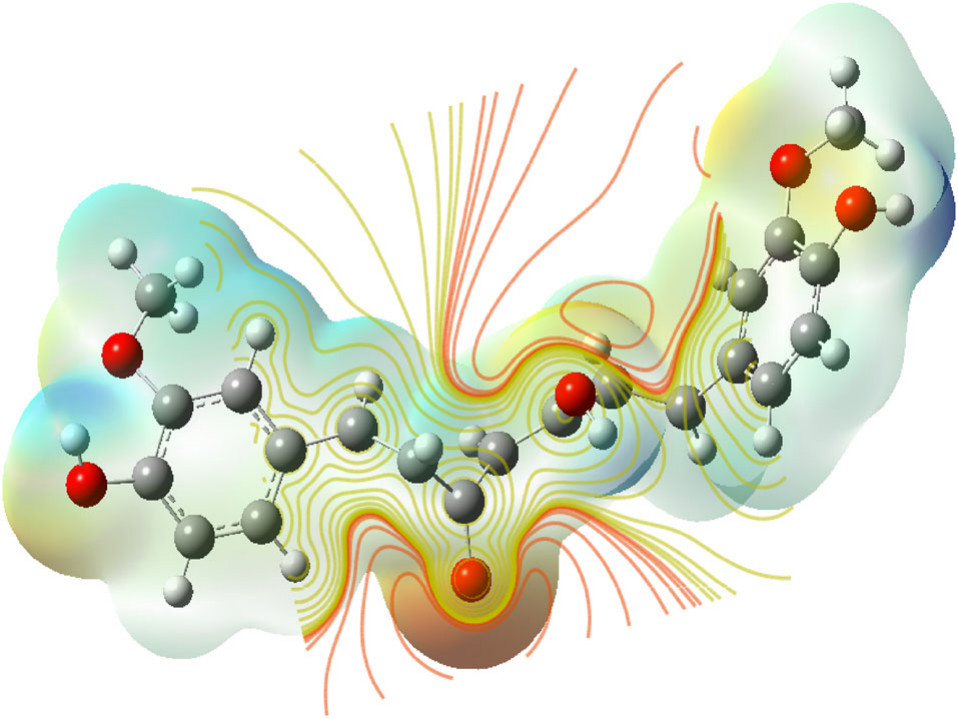
Electron density maps of curcumin

**Fig. 16:**
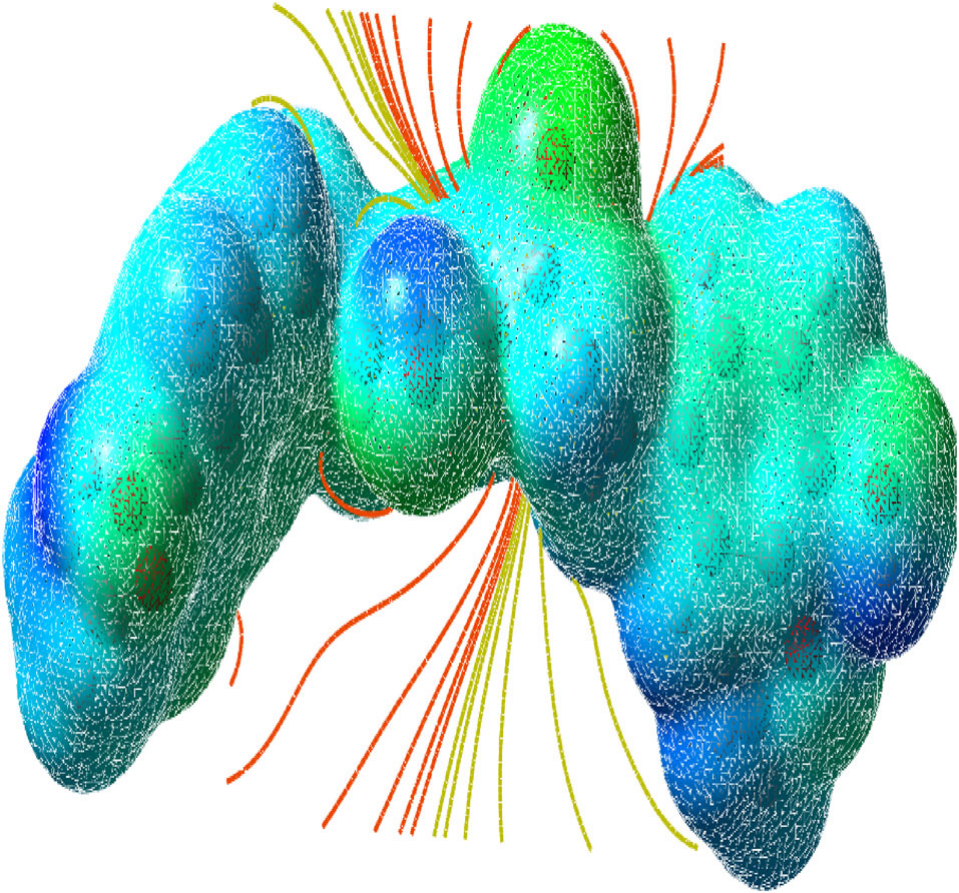
The molecular electrostatic potential surface of curcumin

The molecular electrostatic potential is related to the electronic density and is a very useful descriptor for determining the sites for electrophilic and nucleophilic reactions as well as hydrogen bonding interactions [36]. To predict reactive sites of electrophilic or nucleophilic attack for the investigated molecule, the MEP at the B3LYP/6-311++G(d,p) optimized geometry was calculated. The various values of the electrostatic potential at the surface are represented by various colors. The color scheme for the MEP surface is red-electron rich, partially negative charge (electrophilic reactive center); blue-electron deficient, partially positive charge; light blue-slightly electron deficient region (nucleophilic reactive center); yellow-slightly electron rich region; green- neutral, respectively. The potential increases in the order red < orange < yellow < green < cyan < blue. It can be seen that the negative regions are mainly over the N atoms. The negative (red and yellow) regions of the MEP are related to electrophilic reactivity and the positive (blue) regions to nucleophilic reactivity, as shown in Figs. 14, 15 and 16. As can be seen from the figure, this molecule has several possible sites, C and O atoms for electrophilic attack. The importance of MESP lies in the fact that it simultaneously displays molecular size, shape as well as positive, negative and neutral electrostatic potential regions in terms of color grading and is very useful in research of molecular structure with its physiochemical property relationship.

### Thermodynamic Properties

**Fig. 17:**
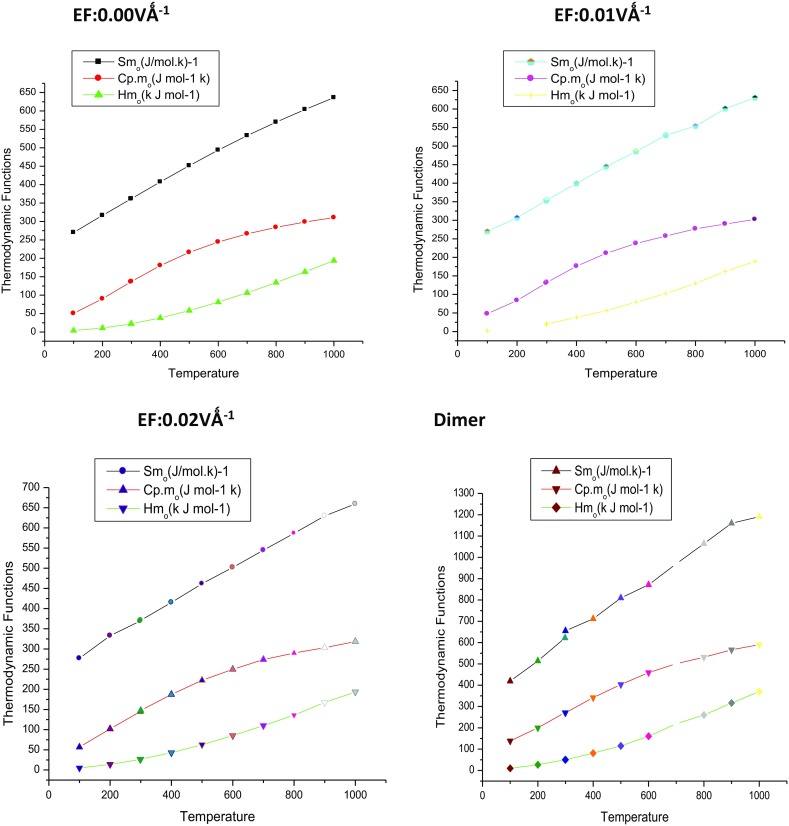
Correlation graphic of entropy, enthalpy and heat capacity with various temperature of Curcumin

Standard statistical thermodynamics function, heat capacity $\left( {{\text{C}}_{\text{p,m}}^{0} } \right)$, entropy $\left( {{\text{S}}_{\text{m}}^{0} } \right)$ and enthalpy changes $\left( {{\text{H}}_{\text{m}}^{0} } \right)$ (monomer and dimer) were computed at B3LYP/6-311++G(d,p) basis set by using perl script THERMO.PL [37] and are listed in Table 10. Thermodynamic functions are all values increasing with temperature ranging from 100 to 1000 K due to the fact that the molecular vibrations intensities increase with temperature. The correlation equation among heat capacities, entropies, enthalpy changes with temperatures were fitted by quadratic formulas and the corresponding fitting factors (R^2^) these thermodynamic properties are respectively. The correlations plot of those shown in Fig. 17. The thermodynamic correlation fitting equation is follows: Moreover, the thermodynamic properties indicate that the curcumin molecule is more reactive and more polar. The correlation graph for zero (monomer and dimer) applied EFs are shown in Fig. 17 and the corresponding fitting equations are as follows:

**Table 10:** Thermodynamic properties at different temperatures at the B3LYP/6-311++G(d,p) level for curcumin

T (K)	$\left( {{\text{S}}_{\text{m}}^{0} } \right)$ (J mol k)^−1^				$\left( {{\text{C}}_{\text{p,m}}^{0} } \right)$ (J mol^−1^k)				$\left( {{\text{H}}_{\text{m}}^{0} } \right)$ (kJ mol^−1^)			
	0.00 VÅ^−1^	0.01 VÅ^−1^	0.02 VÅ^−1^	Dimer	0.00 VÅ^−1^	0.01 VÅ^−1^	0.02 VÅ^−1^	Dimer	0.00 VÅ^−1^	0.01 VÅ^−1^	0.02 VÅ^−1^	Dimer
100.00	270.12	269.90	276.76	419.15	50.50	47.83	56.71	138.29	3.92	2.70	4.98	9.91
200.00	316.45	306.76	332.99	513.76	90.18	83.70	101.72	199.91	10.83	09.83	13.88	26.72
298.15	361.00	353.23	369.12	622.09	136.12	130.66	144.73	271.57	21.93	19.64	25.91	49.81
300.00	361.84	354.99	371.00	655.34	136.98	132.39	147.49	270.96	22.18	20.89	26.77	50.31
400.00	407.34	399.12	415.23	711.89	180.33	176.25	187.15	341.65	38.10	37.90	42.90	81.30
500.00	451.56	444.65	461.78	809.44	216.02	210.90	222.20	403.09	57.98	56.61	62.58	115.13
600.00	493.54	485.22	502.18	871.40	244.15	237.43	249.59	459.36	81.05	79.23	85.12	160.74
700.00	532.90	529.13	544.67	969.48	266.30	257.21	273.72	500.80	106.62	102.26	109.92	219.07
800.00	569.66	553.90	586.61	1060.12	284.04	276.75	289.94	531.49	134.16	130.08	136.03	260.24

For 0.00 VÅ^−1^

$$ {\text{S}}_{\text{m}}^{0} \; = \;215.23396\; + \;0.64772\;{\text{T}}\; - \;0.00338\; \times \;10^{ - 5} \;{\text{T}}^{2} \;\left( {{\text{R}}^{2} = \, 0.99884} \right) $$

$$ {\text{C}}_{{{\text{p}},{\text{m}}}}^{0} \; = \;8.94698\; + \;0.58974\;{\text{T}}\; + \;3.63885\; \times \;10^{ - 5} \;{\text{T}}^{2} \;\left( {{\text{R}}^{2} = \, 0.99886} \right) $$

$$ {\text{H}}_{\text{m}}^{0} \; = \; - 2.8146\; + \;1 \, .51434\;{\text{T}}\; - \;2.72041\; \times \;10^{ - 5} \;{\text{T}}^{2} \;\left( {{\text{R}}^{2} = \, 0.9994} \right) $$

For 0.01 VÅ^−1^

$$ {\text{S}}_{\text{m}}^{0} \; = \;225.93312\; + \;0.31634\,{\text{T}}\; + \;8.89829\; \times \;10^{ - 5} \;{\text{T}}^{2} \;\left( {{\text{R}}^{2} = \, 0.99906} \right) $$

$$ {\text{C}}_{{{\text{p}},{\text{m}}}}^{0} \; = \;1.61101\; - \;6.77441\;{\text{T}}\; - \;0.06719\; \times \;10^{ - 4} \;{\text{T}}^{2} \;\left( {{\text{R}}^{2} = \, 0.99988} \right) $$

$$ {\text{H}}_{\text{m}}^{0} \; = \;3.98006\; + \;1.41434\;{\text{T}}\; + \;3.09863\; \times \;10^{ - 4} \;{\text{T}}^{2} \;\left( {{\text{R}}^{2} = \, 0.99942} \right) $$

For 0.02 VÅ^−1^

$$ {\text{S}}_{\text{m}}^{0} \; = \;222.93312\; + \;0.73581\;{\text{T}}\; + \;7.55761\; \times \;10^{ - 5} \;{\text{T}}^{2} \;\left( {{\text{R}}^{2} = \, 99928} \right) $$

$$ {\text{C}}_{{{\text{p}},{\text{m}}}}^{0} \; = \;1.61301\; + \;0.5950\;{\text{T}}\; - \;2.2175\; \times \;10^{ - 4} \;{\text{T}}^{2} \;\left( {{\text{R}}^{2} = \, 0.9988} \right) $$

$$ {\text{H}}_{\text{m}}^{0} \; = \; - \;3.48006\; - \;1.4436\;{\text{T}}\; + \;2.009863\; \times \;10^{ - 4} \;{\text{T}}^{2} \;\left( {{\text{R}}^{2} = \, 0.99947} \right) $$

For Dimer

$$ {\text{S}}_{\text{m}}^{0} \; = \;301.40643\; + \;1.53227T\;{-}\;3.62688\; \times \;10^{ - 4} \;{\text{T}}^{2} \;\left( {{\text{R}}^{2} = \, 0.99981} \right) $$

$$ {\text{C}}_{{{\text{p}},{\text{m}}}}^{0} \; = \;6.68149\; + \;5.46266\;{\text{T}}\; - \;0.3397\; \times \;10^{ - 4} \;{\text{T}}^{2} \;\left( {R^{2} = \, 0.99866} \right) $$

$$ {\text{H}}_{\text{m}}^{0} \; = \; - \;5.47053\; + \;2.79217\;{\text{T}}\; - \;0.2397\; \times \;10^{ - 4} \;{\text{T}}^{2} \left( {{\text{R}}^{2} = \, 0.99960} \right) $$

## Conclusion

The stabilities of the curcumin are analysed by PES scan. The vibrational frequencies of the fundamental modes of the compound are precisely assigned and analysed and the theoretical results are compared with the experimental frequencies. The energies of important MO’s, absorption wavelength, oscillator strength and excitation energies of the compound are also determined from TDDFT method and compared with the experimental values. The electric field influence is noticed in HOMO–LUMO gaps of curcumin. The HOMO–LUMO gap extensively decreases from 0.06853 eV to 0.0345 eV for curcumin, respectively as the electric field increases. The first hyperpolarizabilities of curcumin are 3.17 and 3.0450 × 10^−30^ esu, respectively. Hα of curcumin was used in NMR in methonal, acetone and actenitrile. The dependence between the rate constant of tautomerisation of curcumin are studied. Photoluminescence has longer wavelength due to the stabilization of the excited states. MEP study shows that the electrophilic attack takes place at the O position of curcumin.
